# Supplementary material for: Archaeometric studies on rock art at four sites in the northeastern Great Basin of North America
Source: PLoS One. 2022 Jan 26;17(1):e0263189. doi: 10.1371/journal.pone.0263189 (PMC8791535; doi:10.1371/journal.pone.0263189)
Supplement: S1 Fig — (Map services and data available from U.S. Geological Survey, National Geospatial Program). (PDF) [file pone.0263189.s001.pdf]

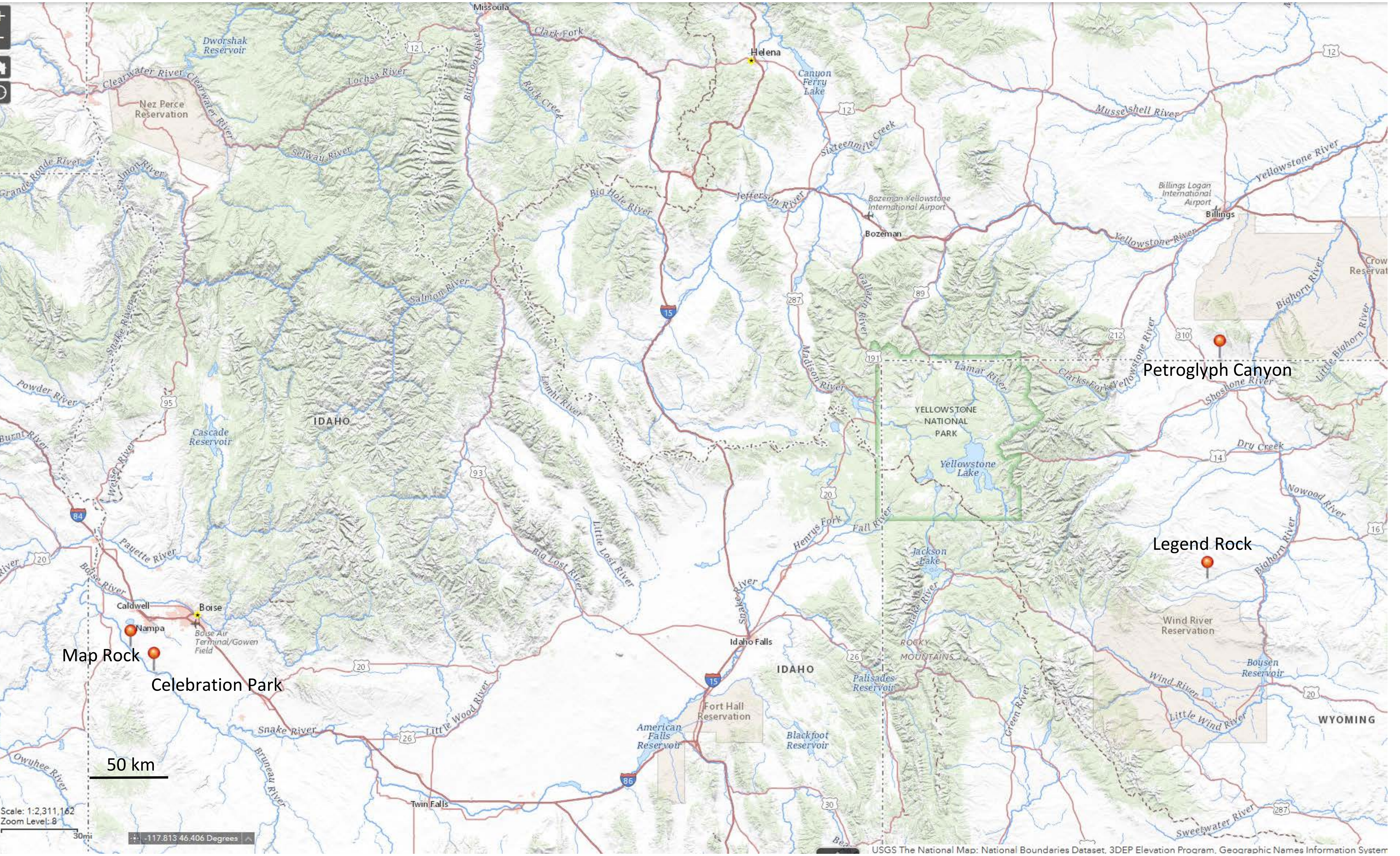

Petroglyph Canyon
Legend Rock
Map Rock
Celebration Park
50 km
Missoula
Helena
Canyon Ferry Lake
Dworshak Reservoir
Nez Perce Reservation
Clearwater River
Lochsa River
Selway River
Bitterroot River
Clark Fork
Rock Creek
Big Hole River
Jefferson River
Sixteenmile Creek
Musselshell River
Yellowstone River
Billings Logan International Airport
Billings
Bozeman Yellowstone International Airport
Bozeman
Crow Reservation
Bighorn River
Little Bighorn River
Gallatin River
Madison River
Salmon River
Grande Ronde River
Snake River
Powder River
Burnt River
Weiser River
Payette River
Boise River
Cascade Reservoir
IDAHO
Lemhi River
Lamar River
Clarks Fork Yellowstone River
Shoshone River
YELLOWSTONE NATIONAL PARK
Yellowstone Lake
Dry Creek
Nowood River
Henrys Fork
Fall River
Jackson Lake
Little Lost River
Big Lost River
Caldwell
Nampa
Boise
Boise Air Terminal/Gowen Field
Idaho Falls
Wind River Reservation
Boysen Reservoir
Wind River
Little Wind River
WYOMING
ROCKY MOUNTAINS
Palisades Reservoir
Green River
Fort Hall Reservation
American Falls Reservoir
Blackfoot Reservoir
Little Wood River
Owyhee River
Bruneau River
Twin Falls
Sweetwater River
Scale: 1:2,311,162
Zoom Level: 8
30mi
-117.813 46.406 Degrees
USGS The National Map: National Boundaries Dataset, 3DEP Elevation Program, Geographic Names Information System
