# Supplementary figures and images for: Archaeometric studies on rock art at four sites in the northeastern Great Basin of North America
Source: PLoS One. 2022 Jan 26;17(1):e0263189. doi: 10.1371/journal.pone.0263189 (PMC8791535; doi:10.1371/journal.pone.0263189)

# CP 2102 & 2226

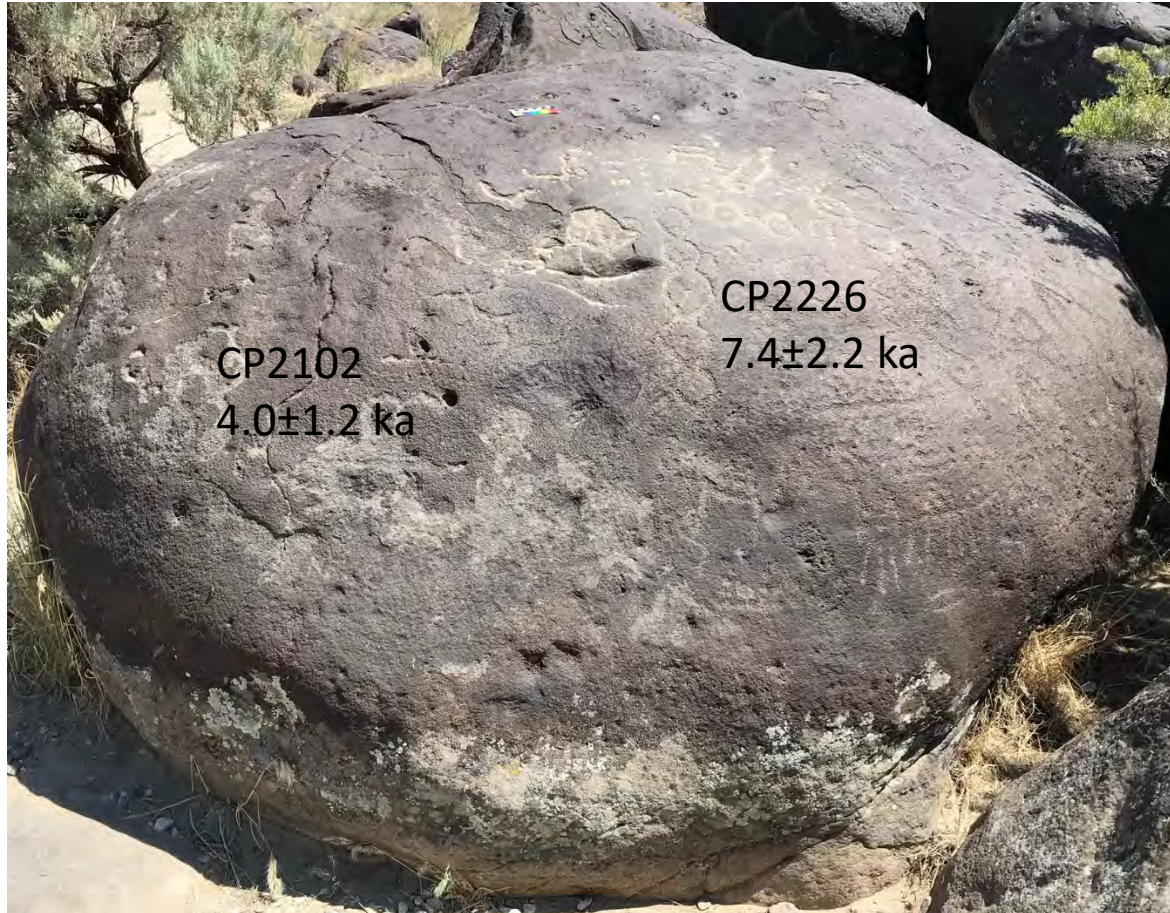

CP 2102

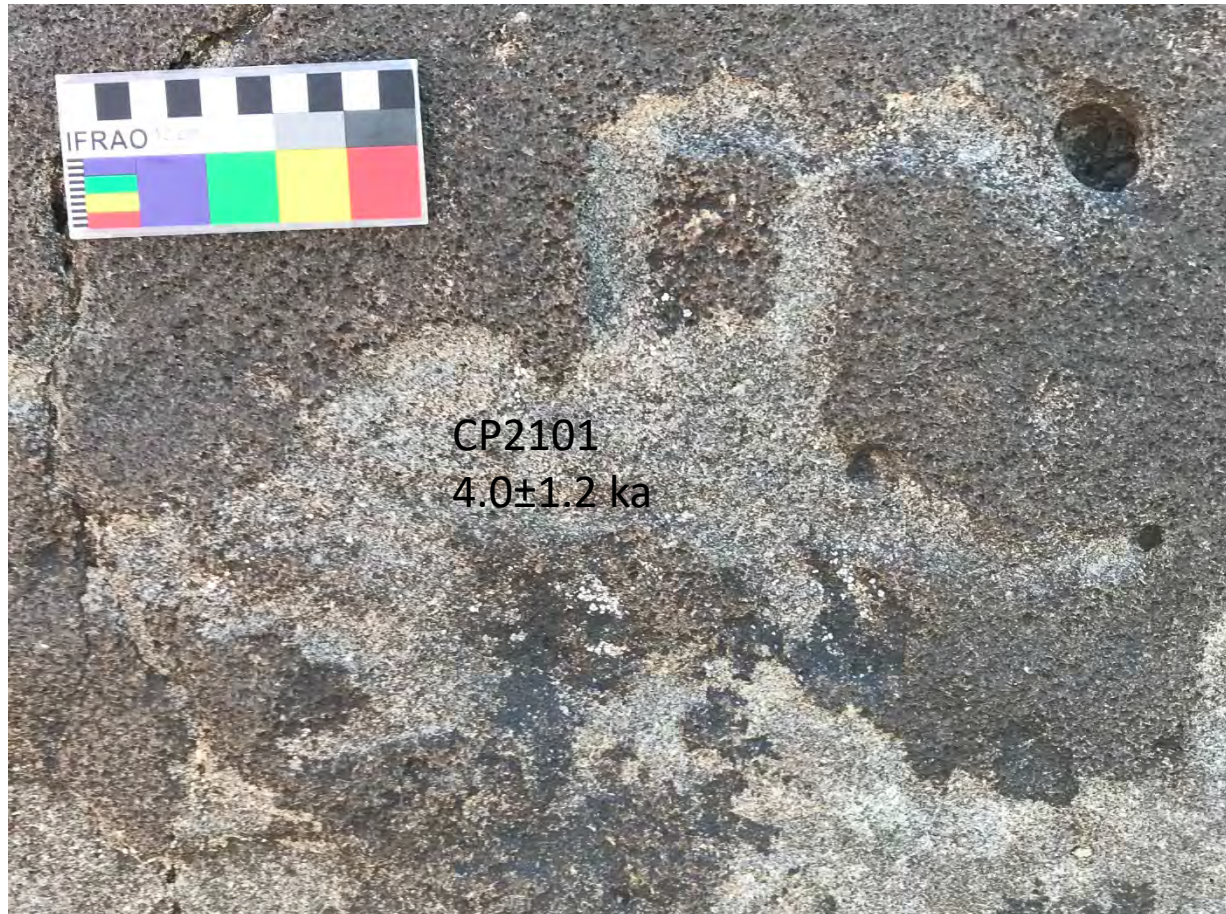

CP 2108

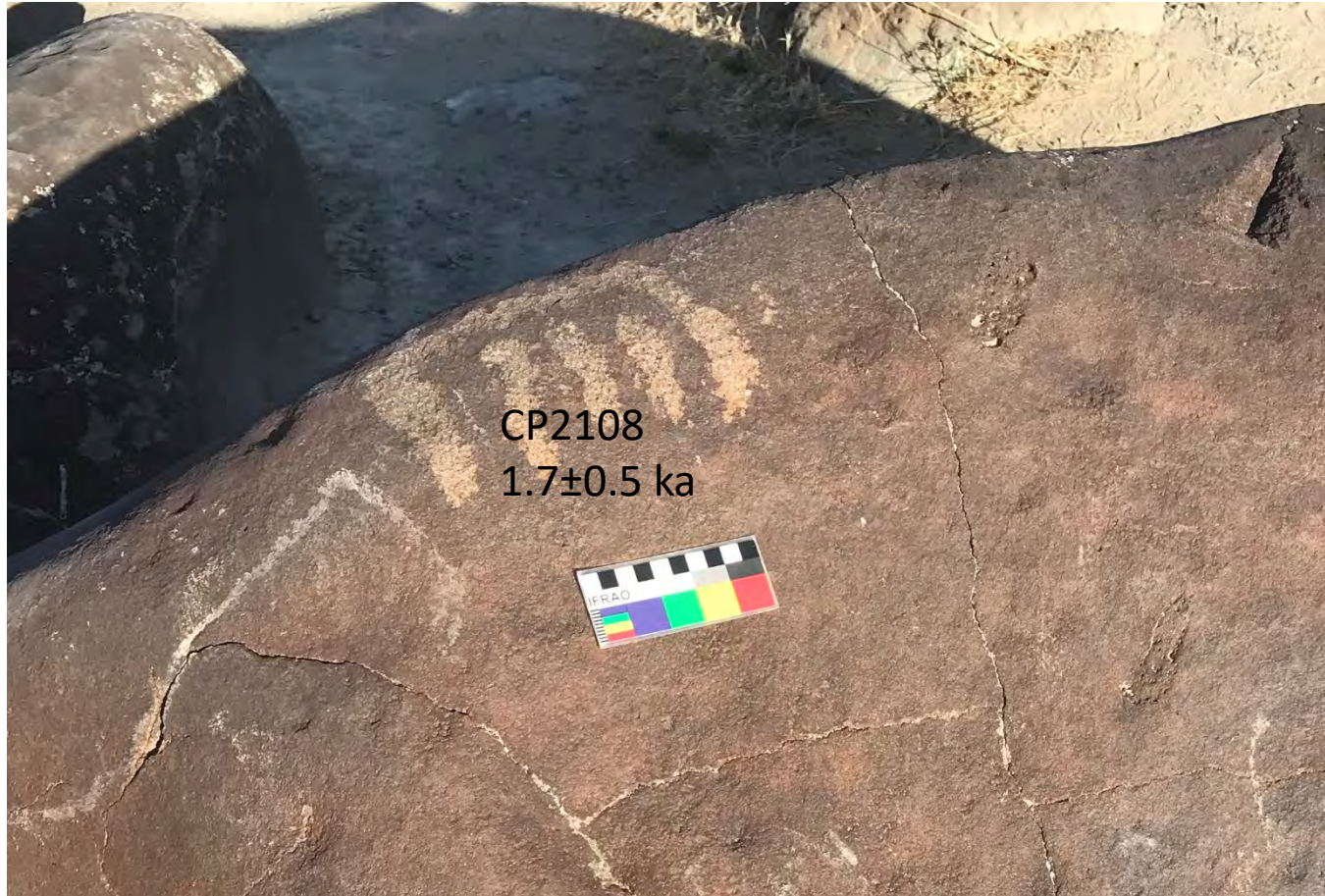

CP 2114

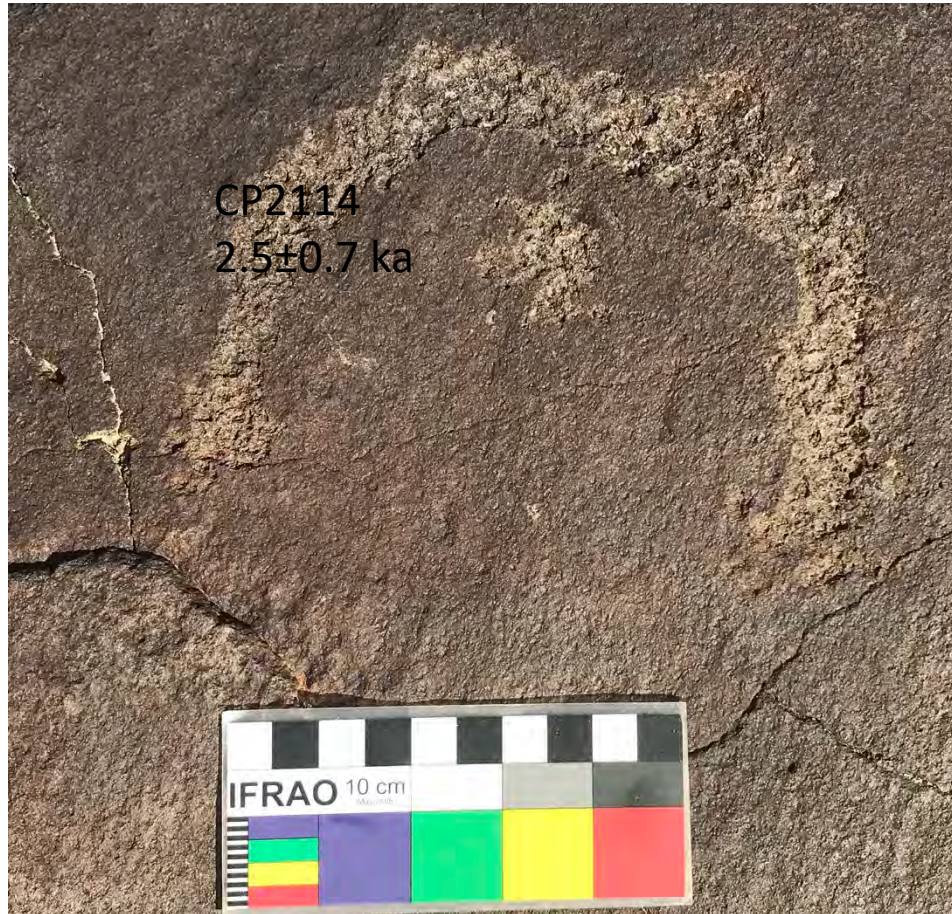

# CP 2120 & 2127

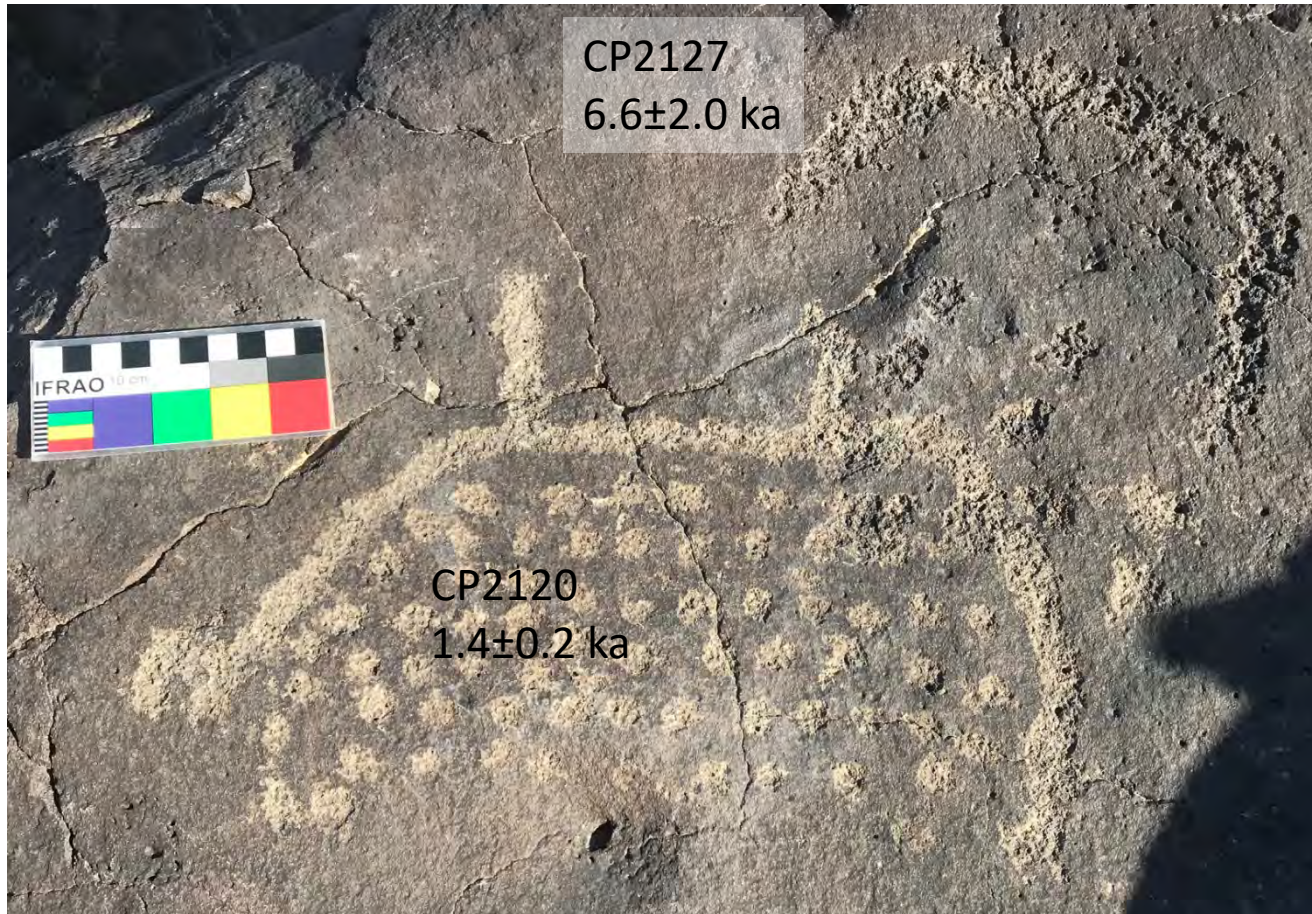

CP 2133

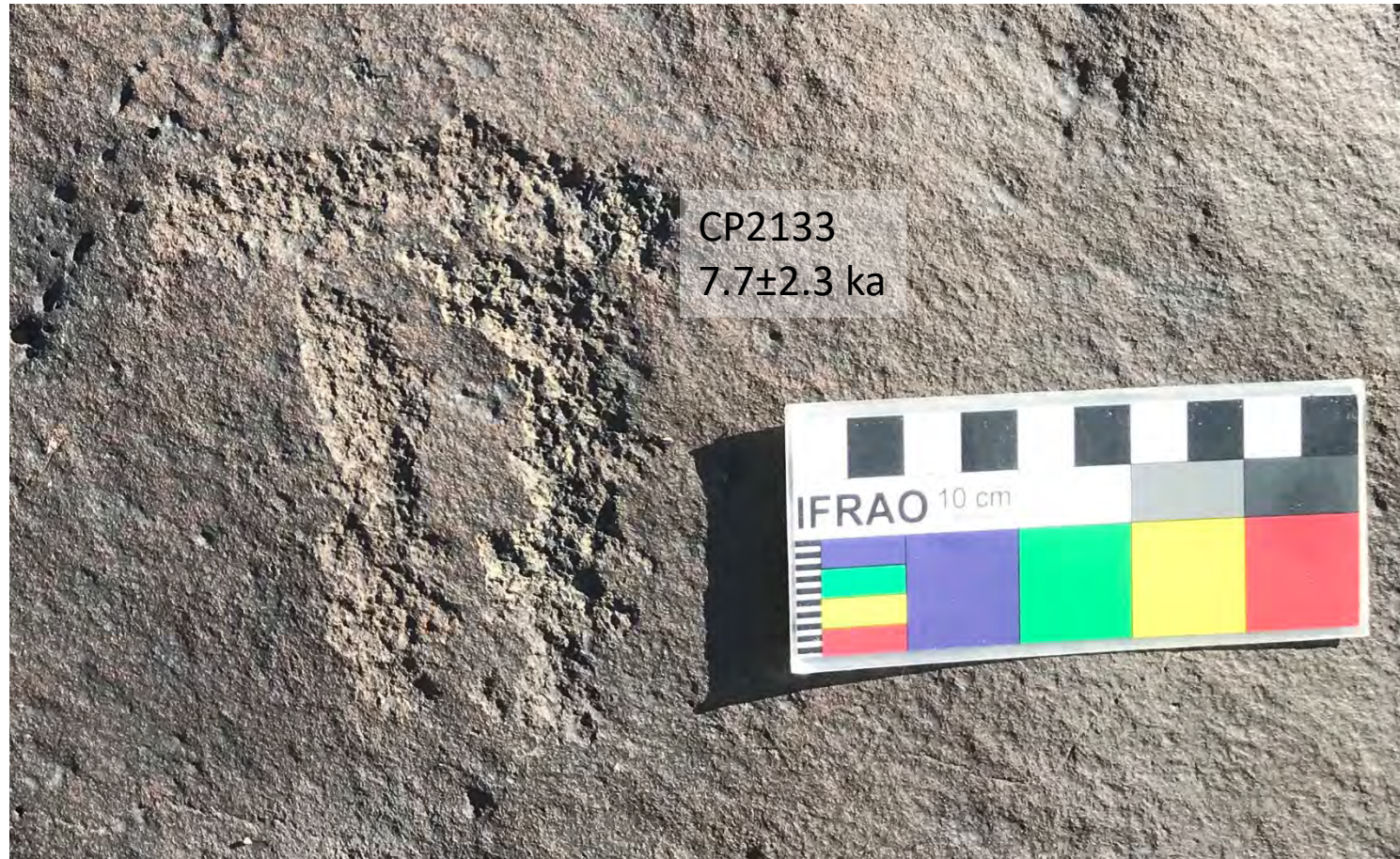

# CP 2140 & 2146

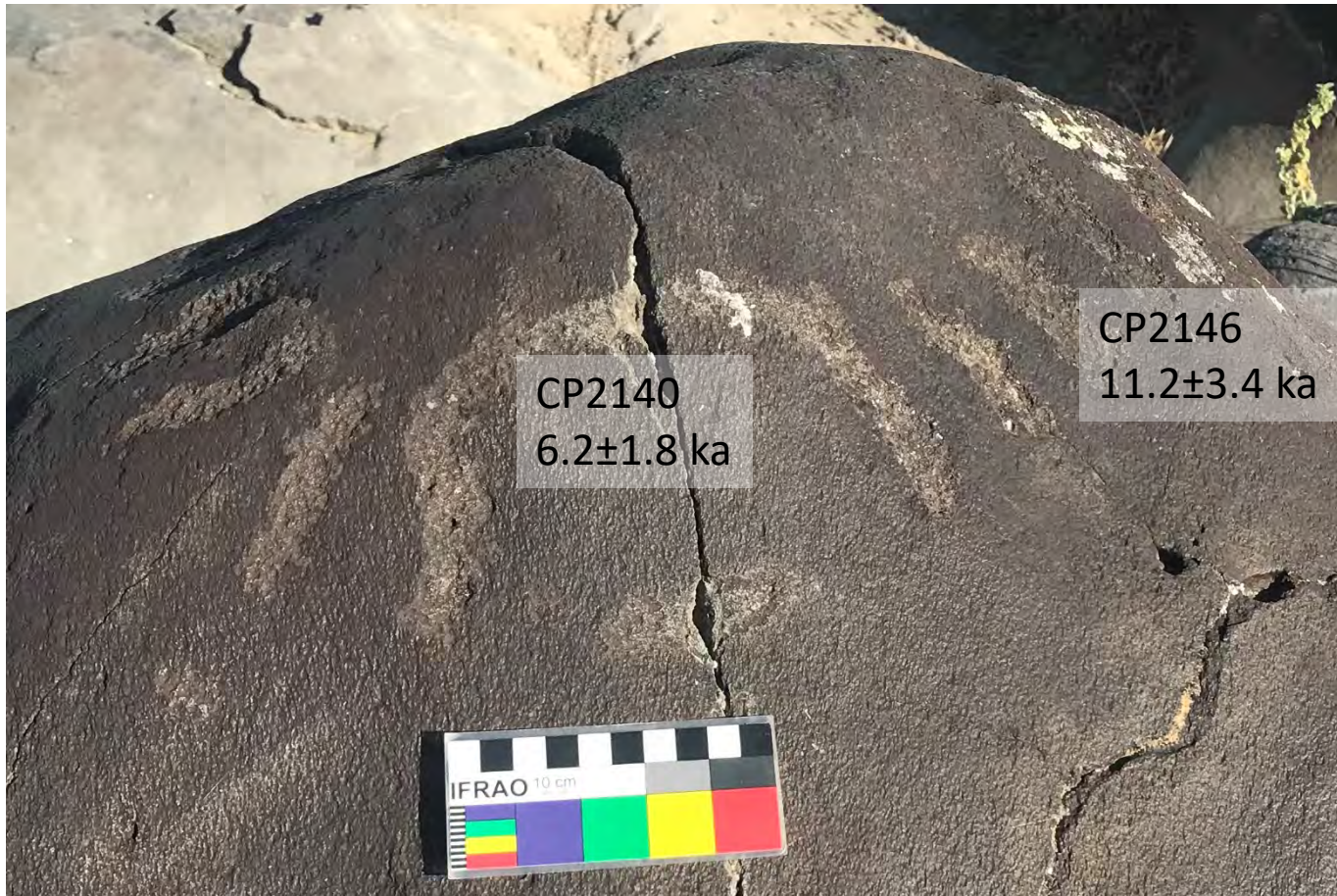

CP 2152

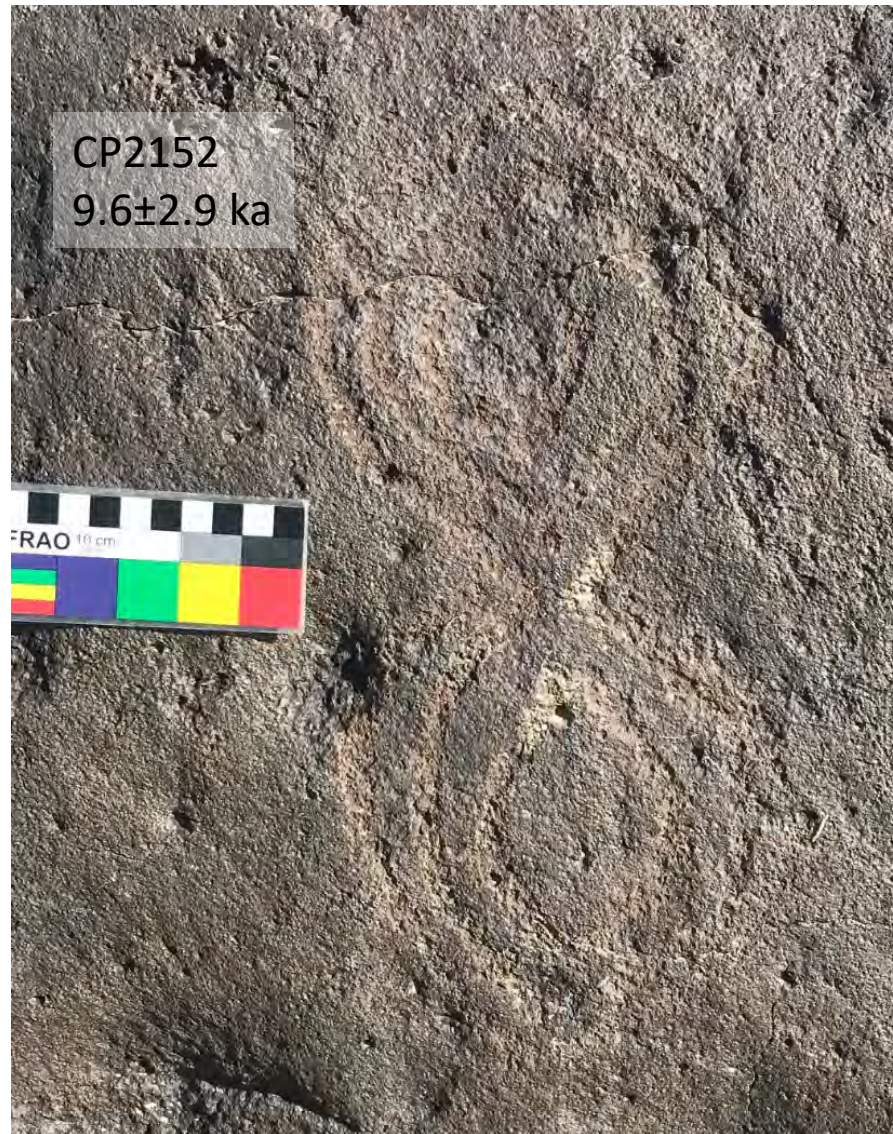

CP 2160

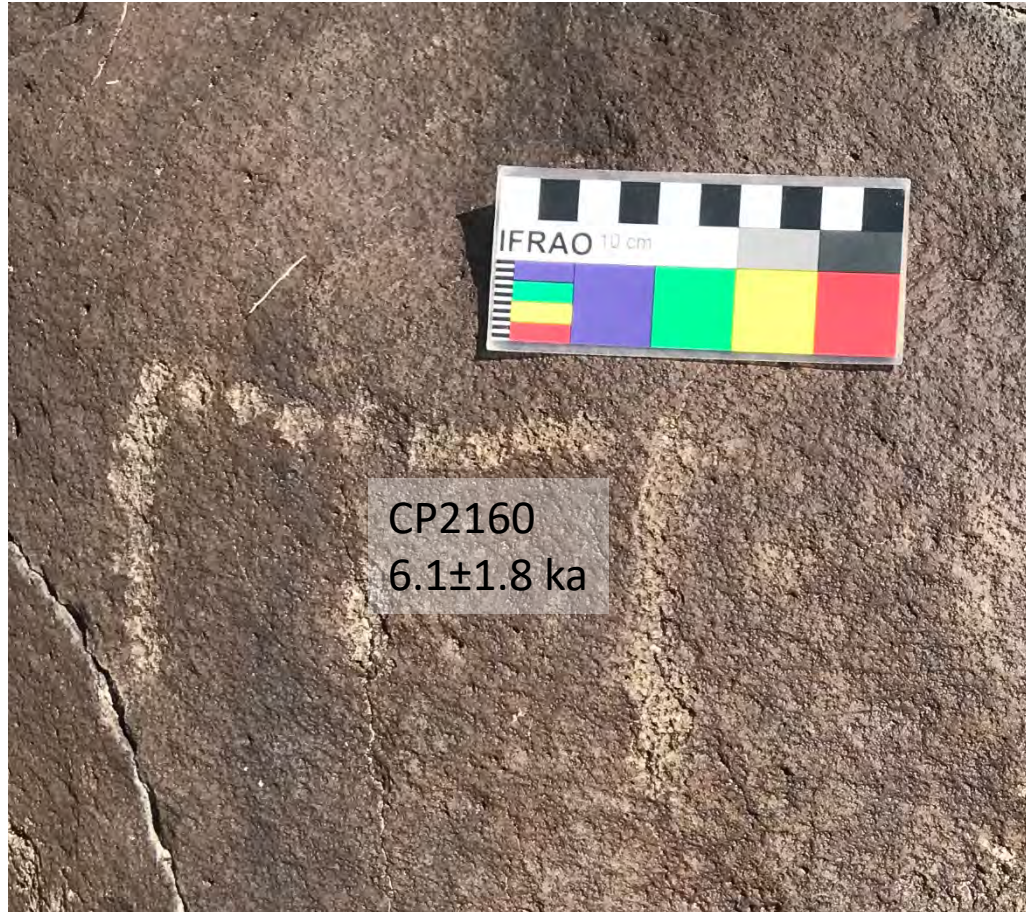

CP2160  
6.1±1.8 ka

CP 2166

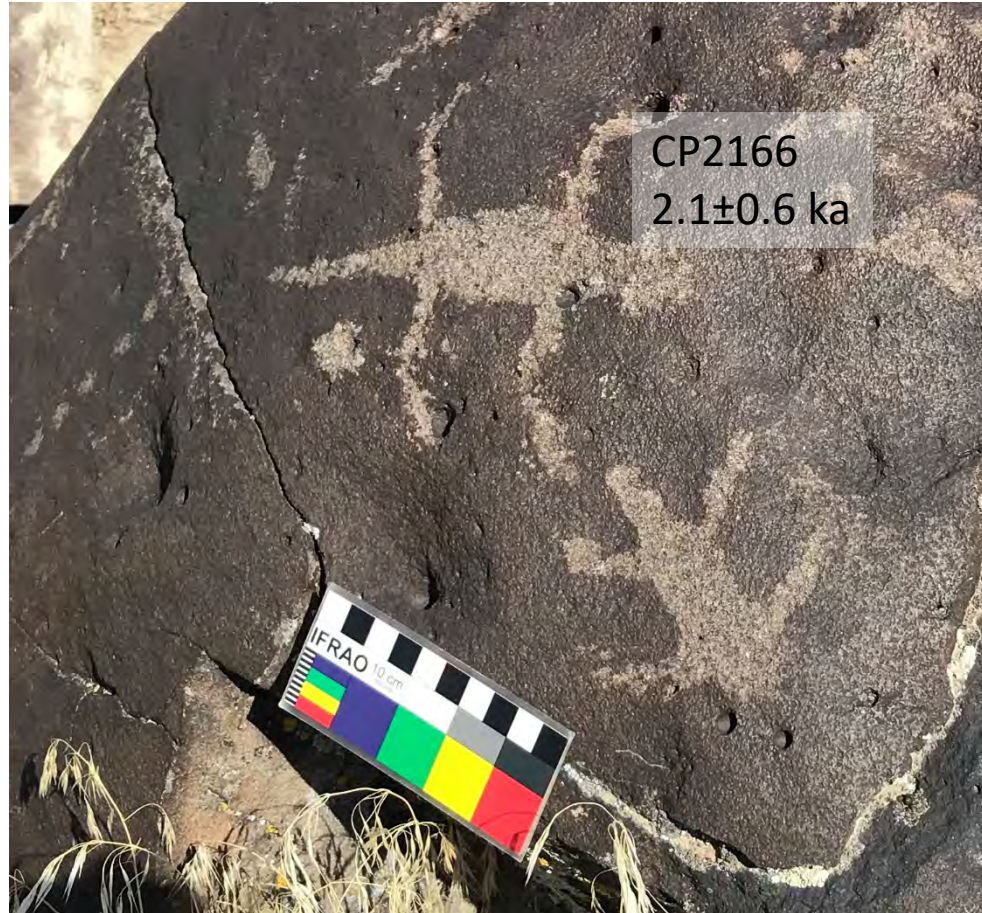

CP 2172

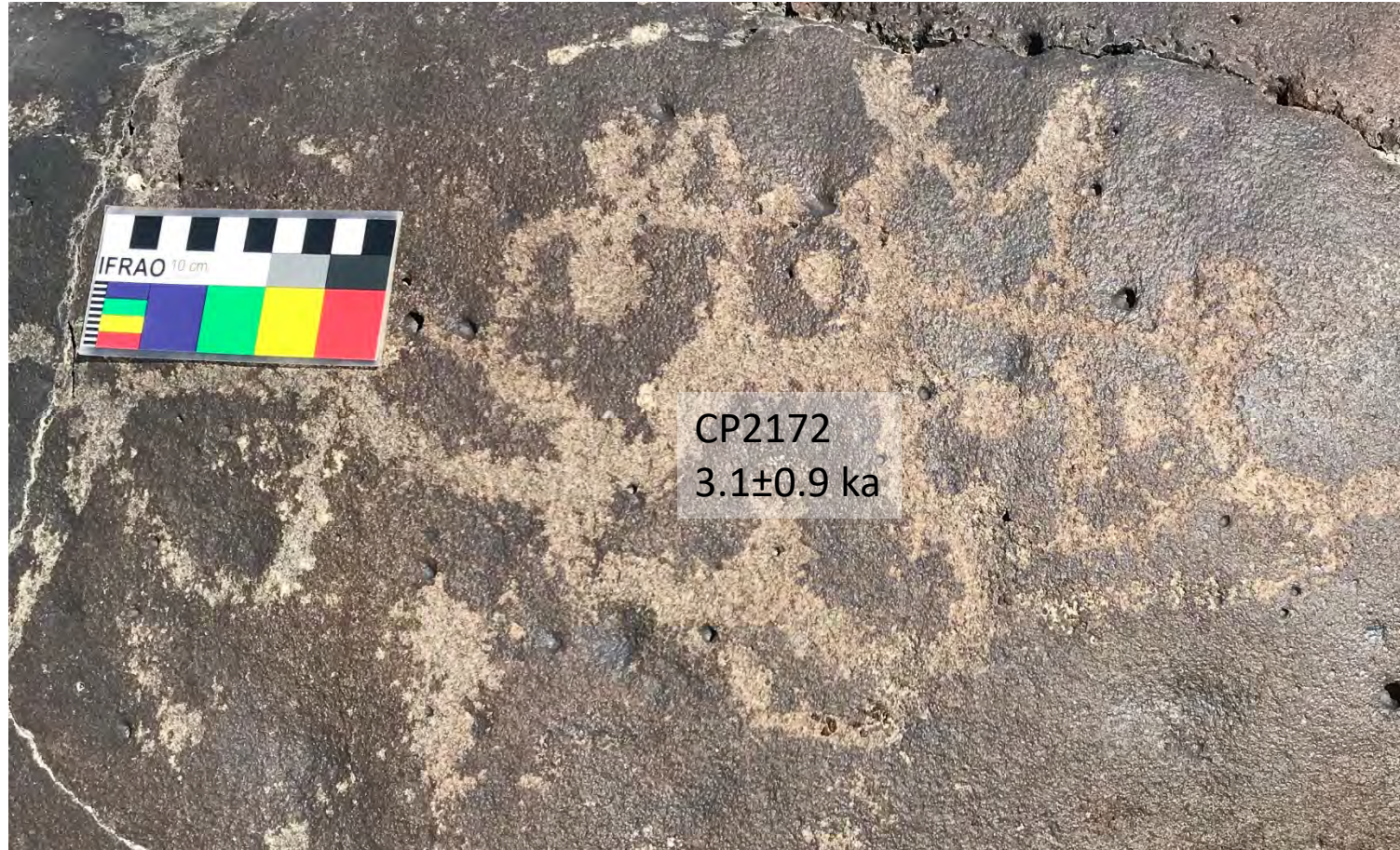

CP 2179

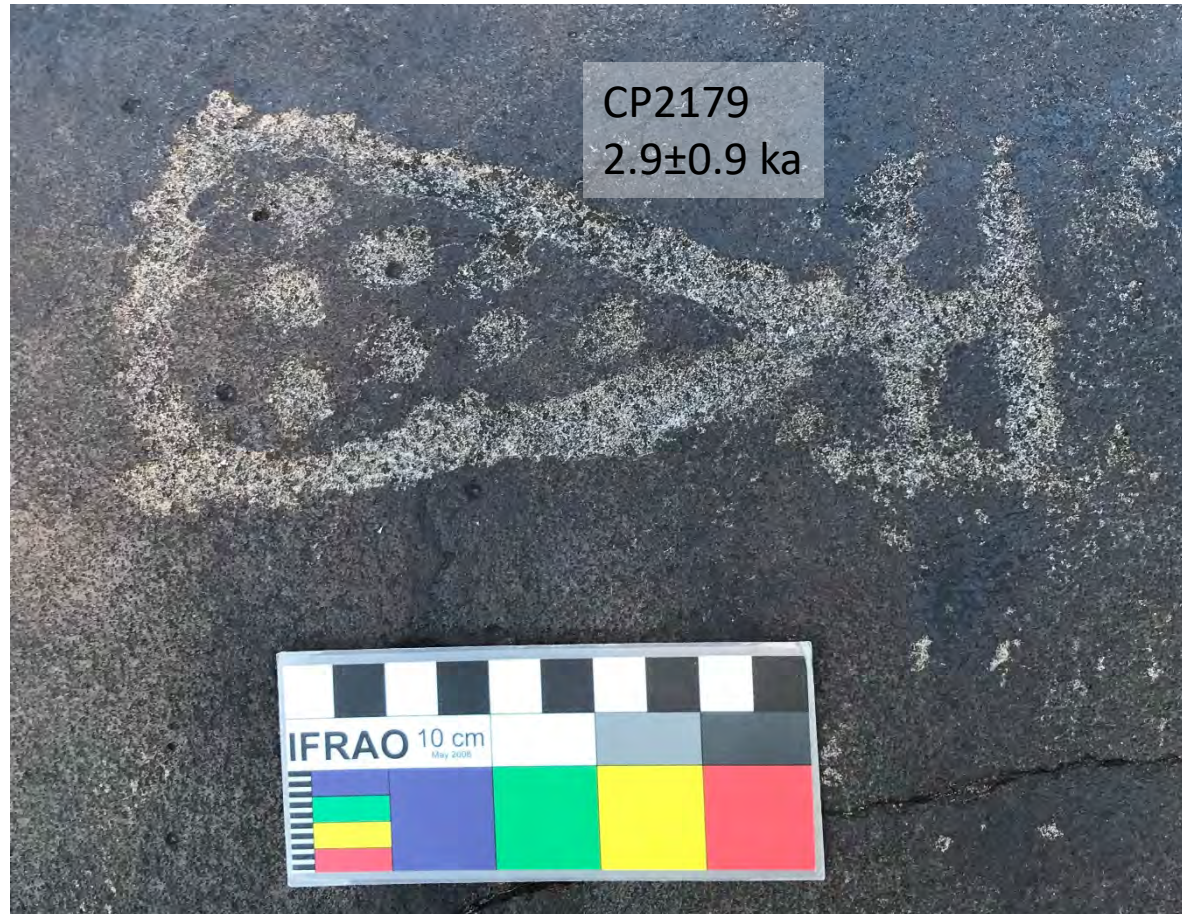

CP 2185

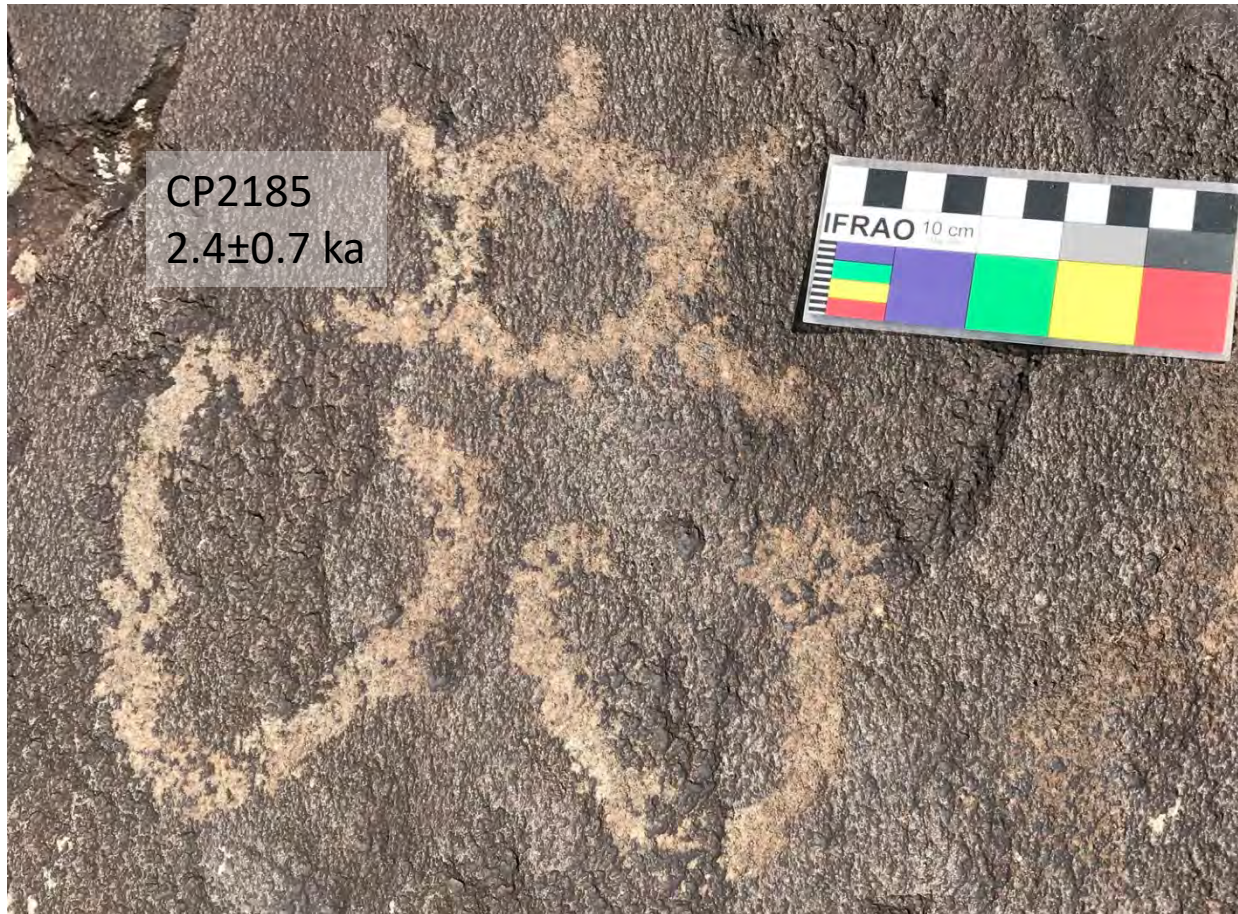

CP 2191

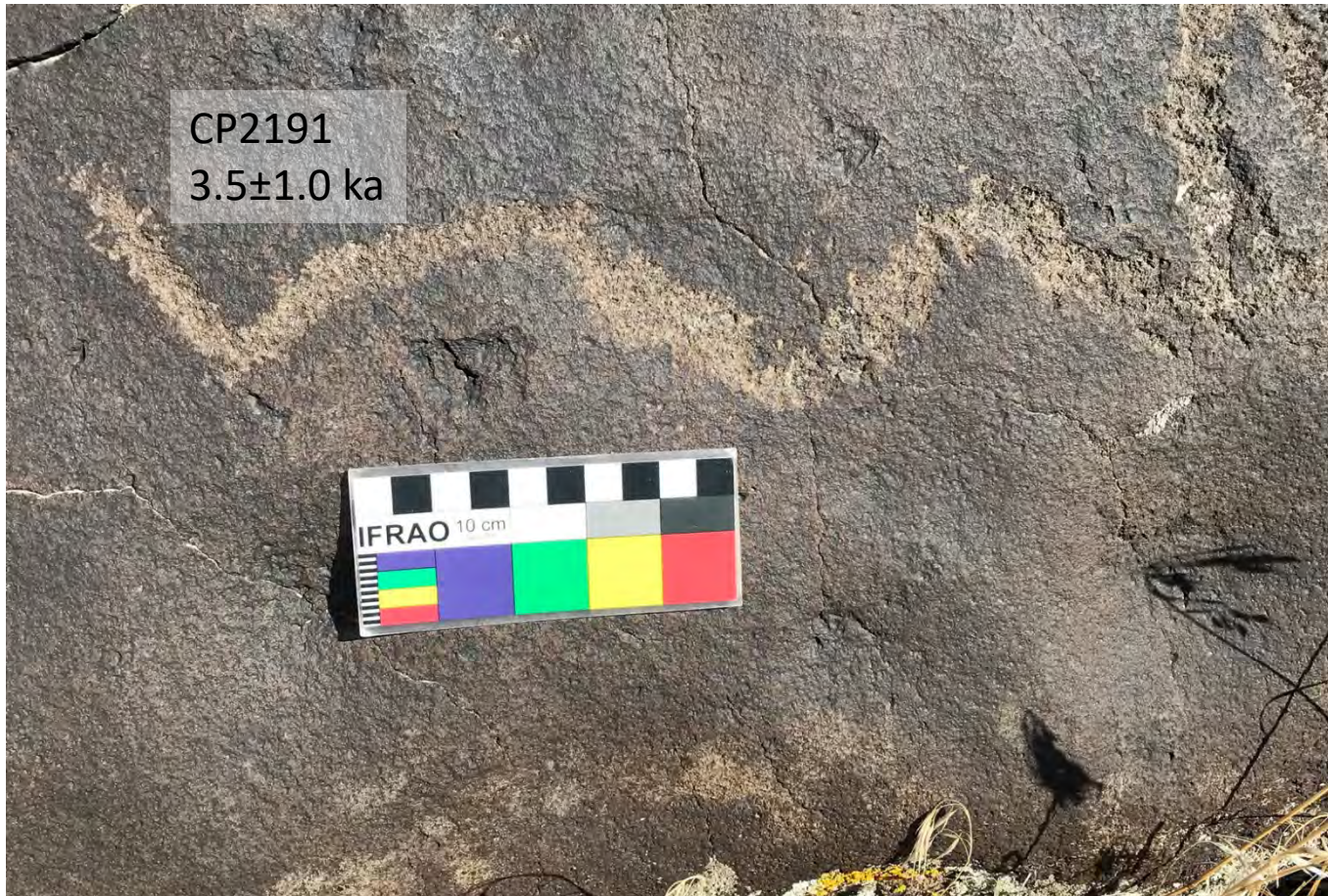

# CP 2198 & 2201

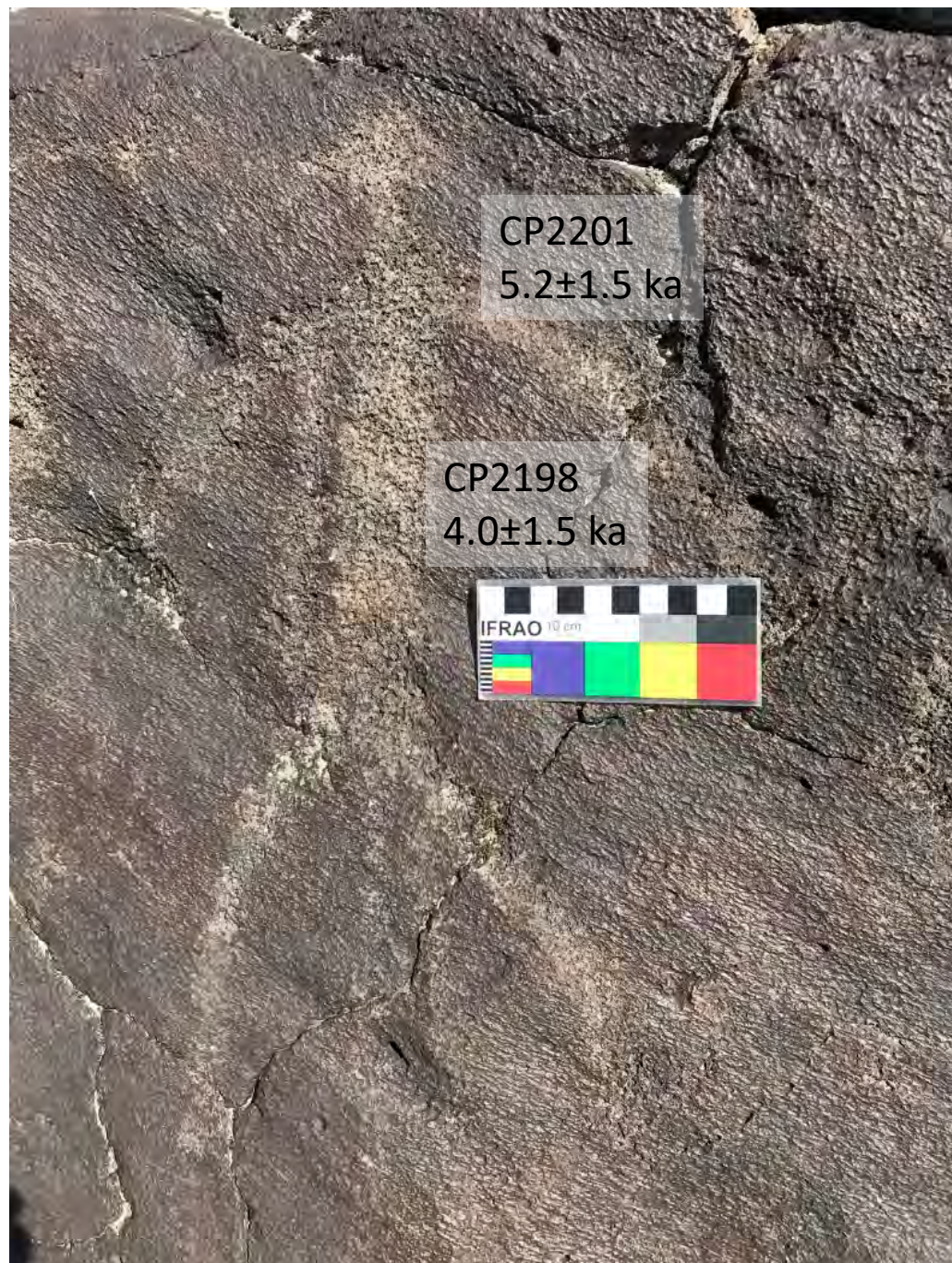

CP 2208

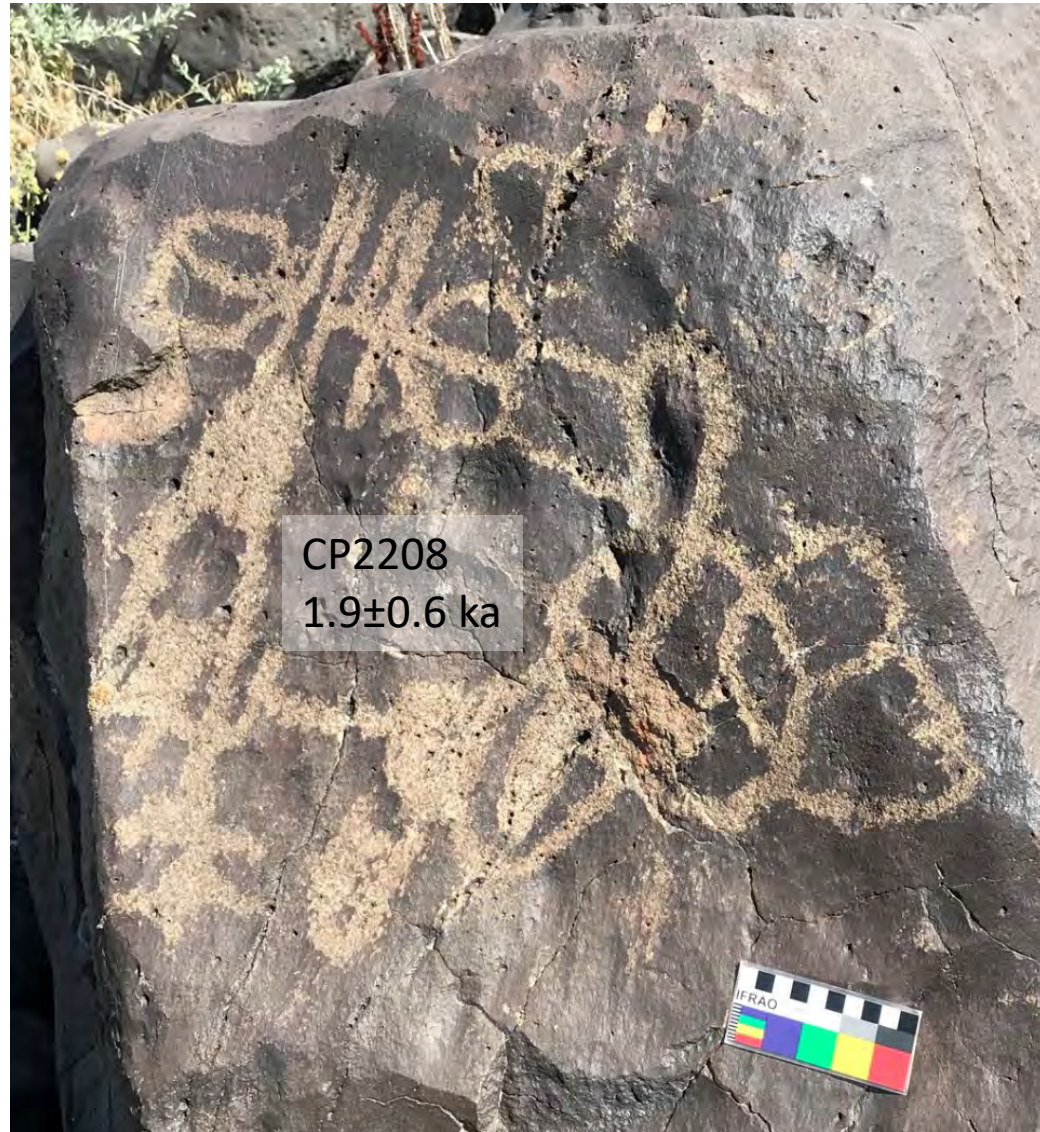

CP 2215

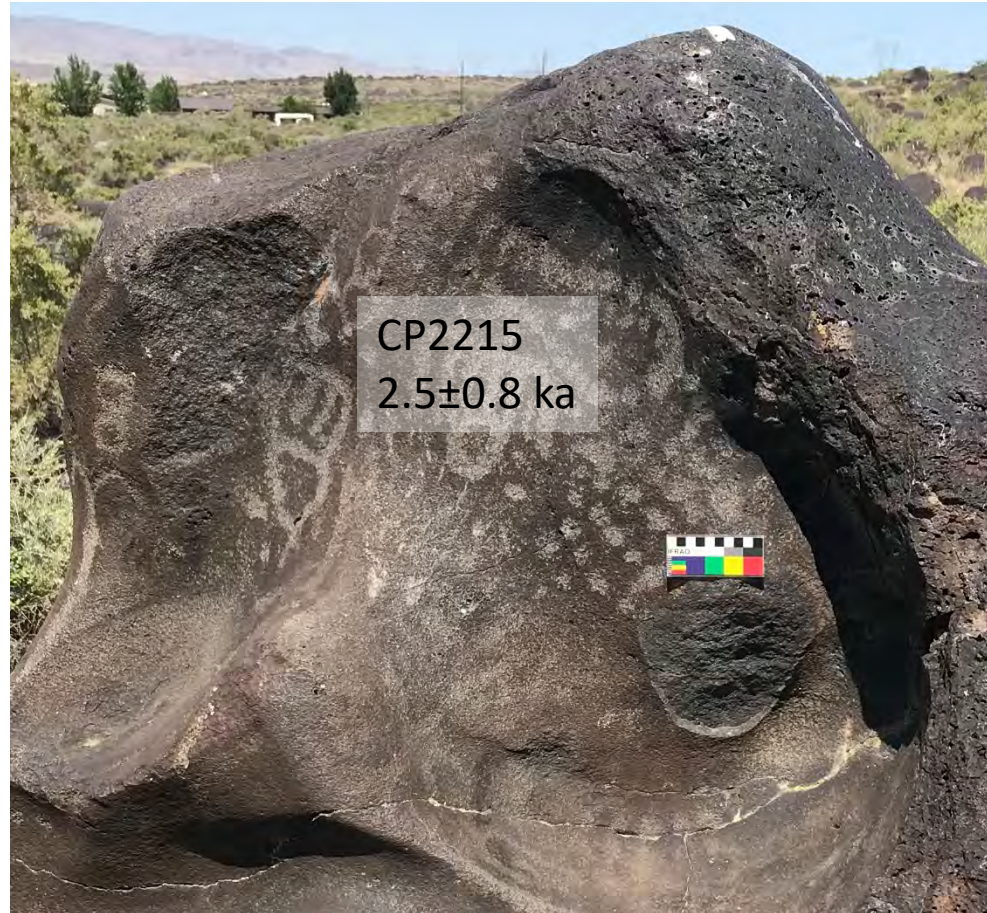

CP 2235

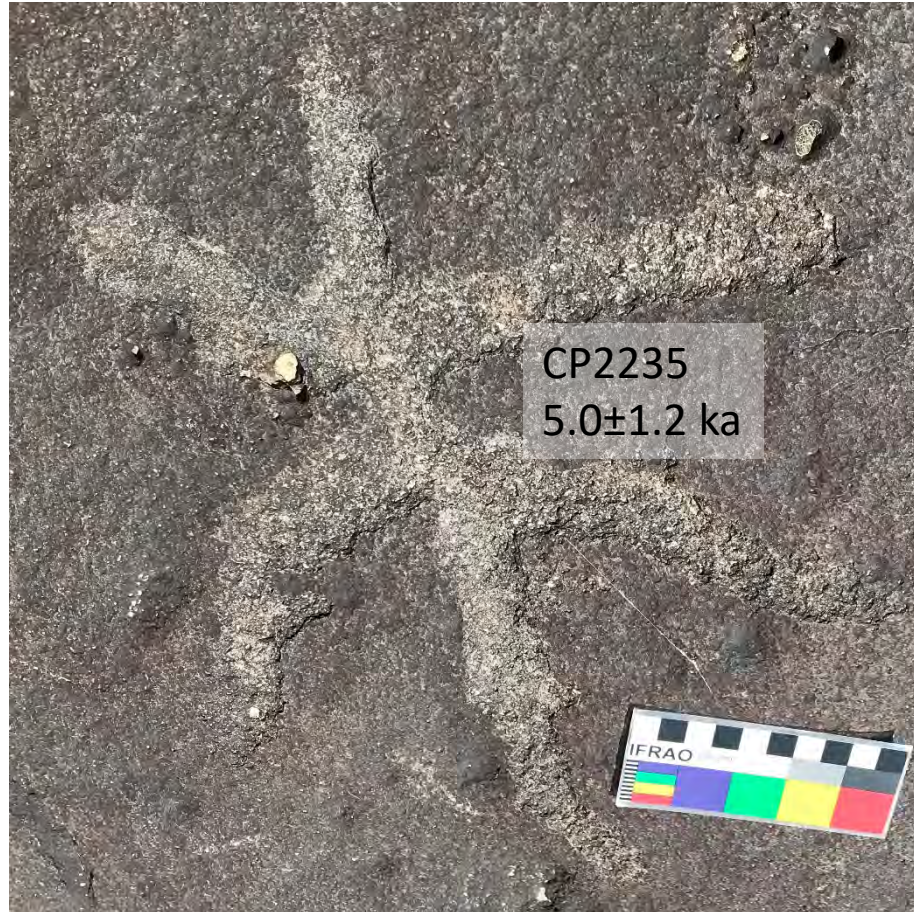

Supplement: S1 File — (PDF) [file pone.0263189.s002.pdf]

# WSW Side

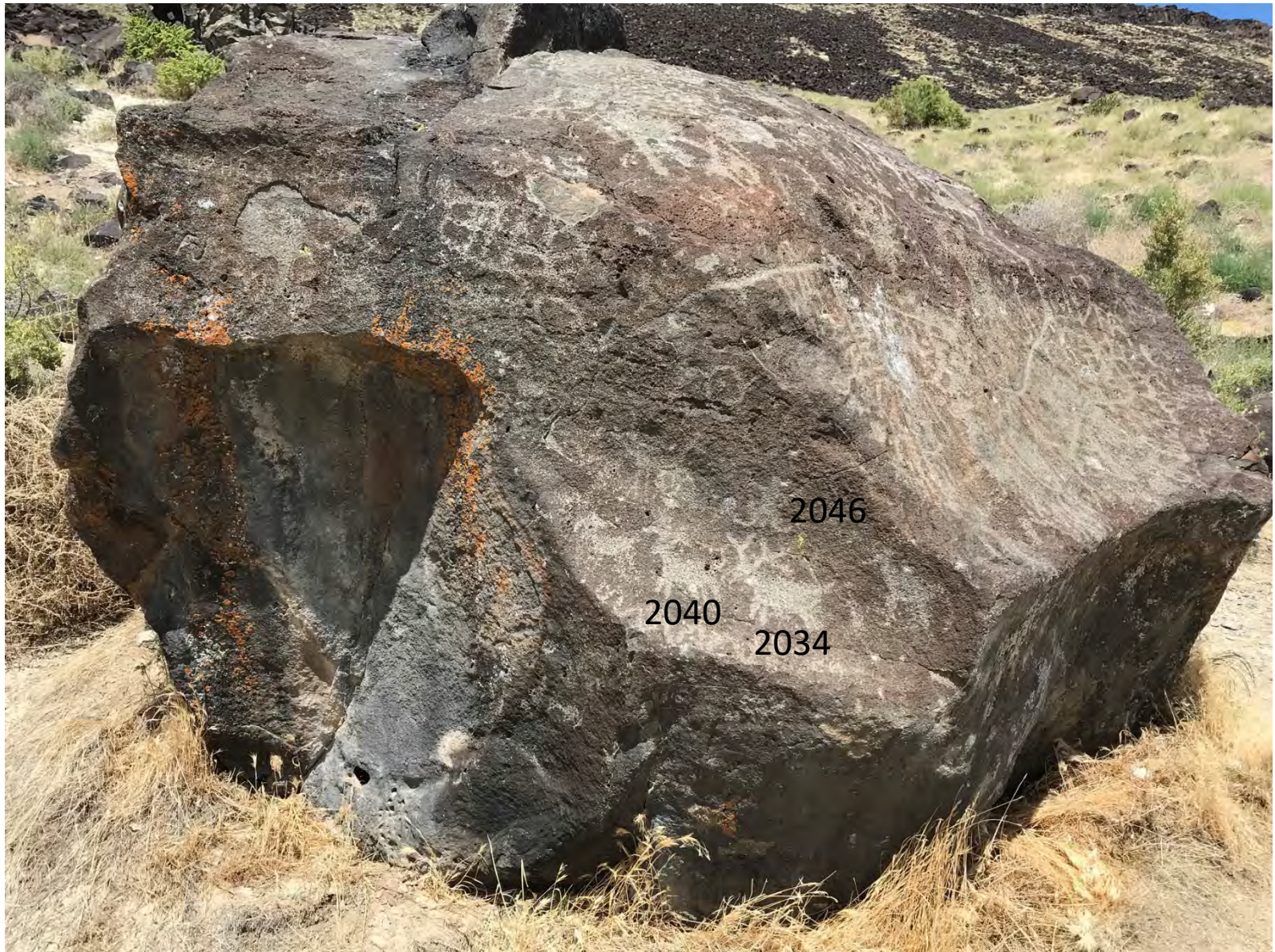

# South Side

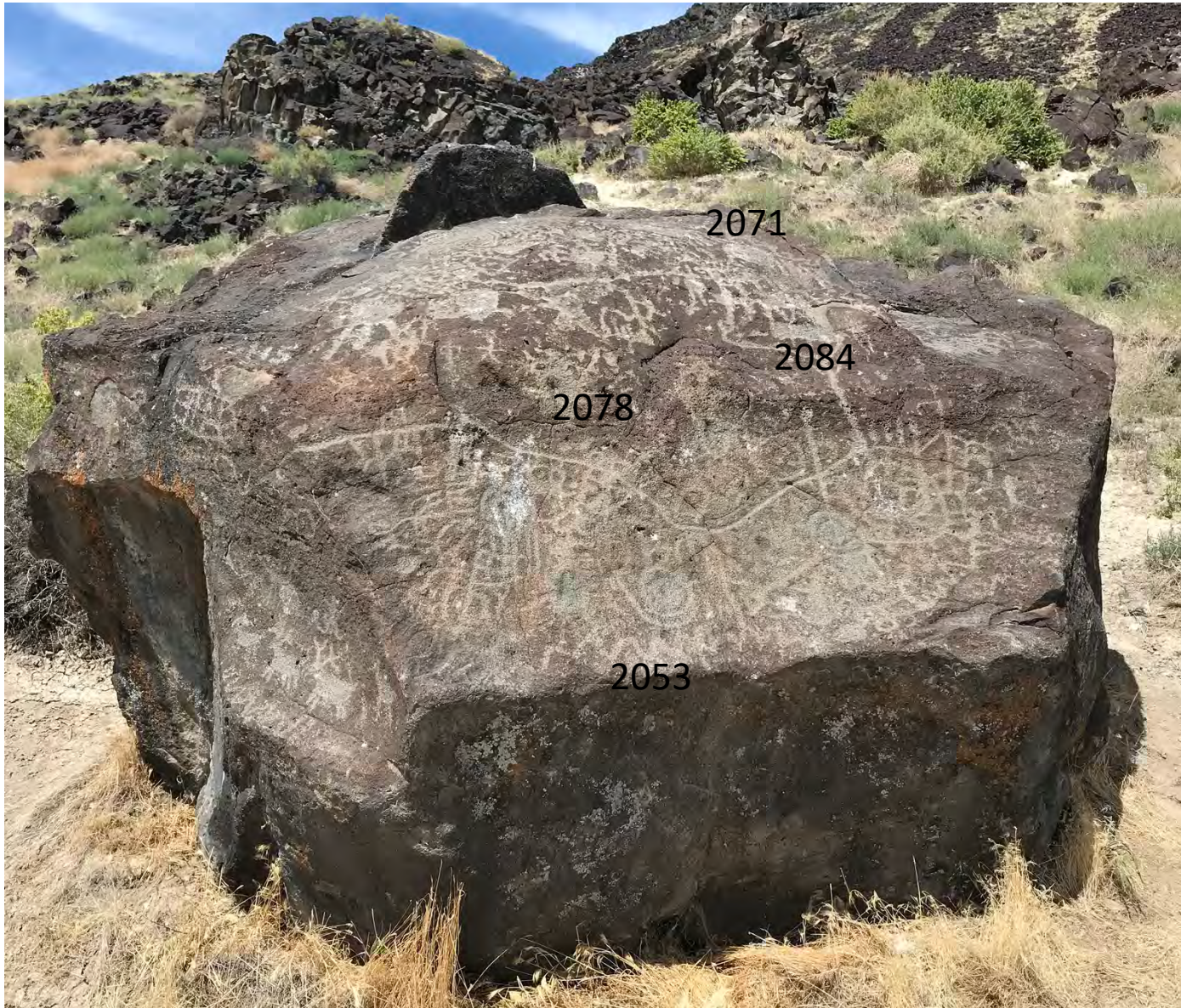

# East Side

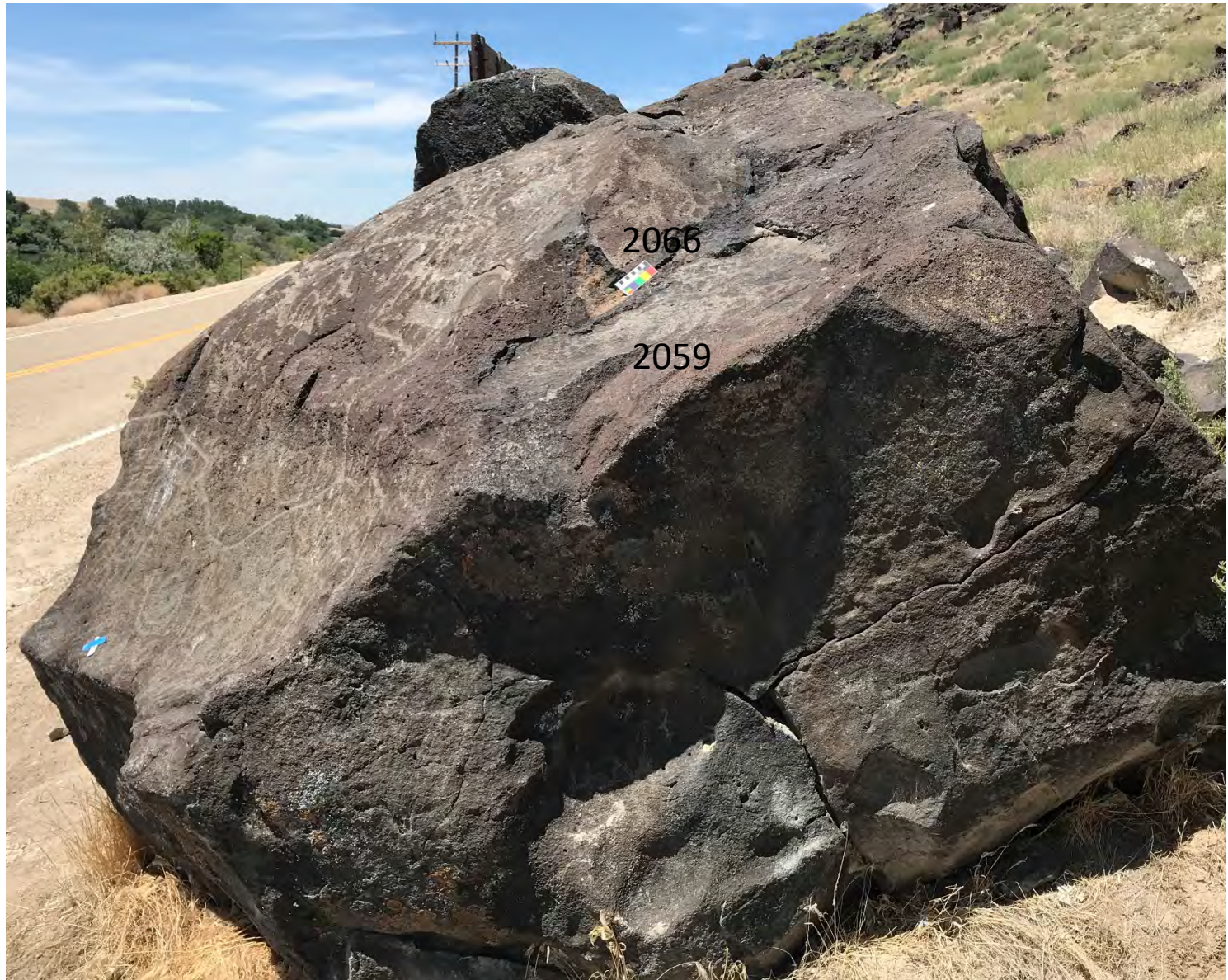

# MR 2034, 2040, 2046

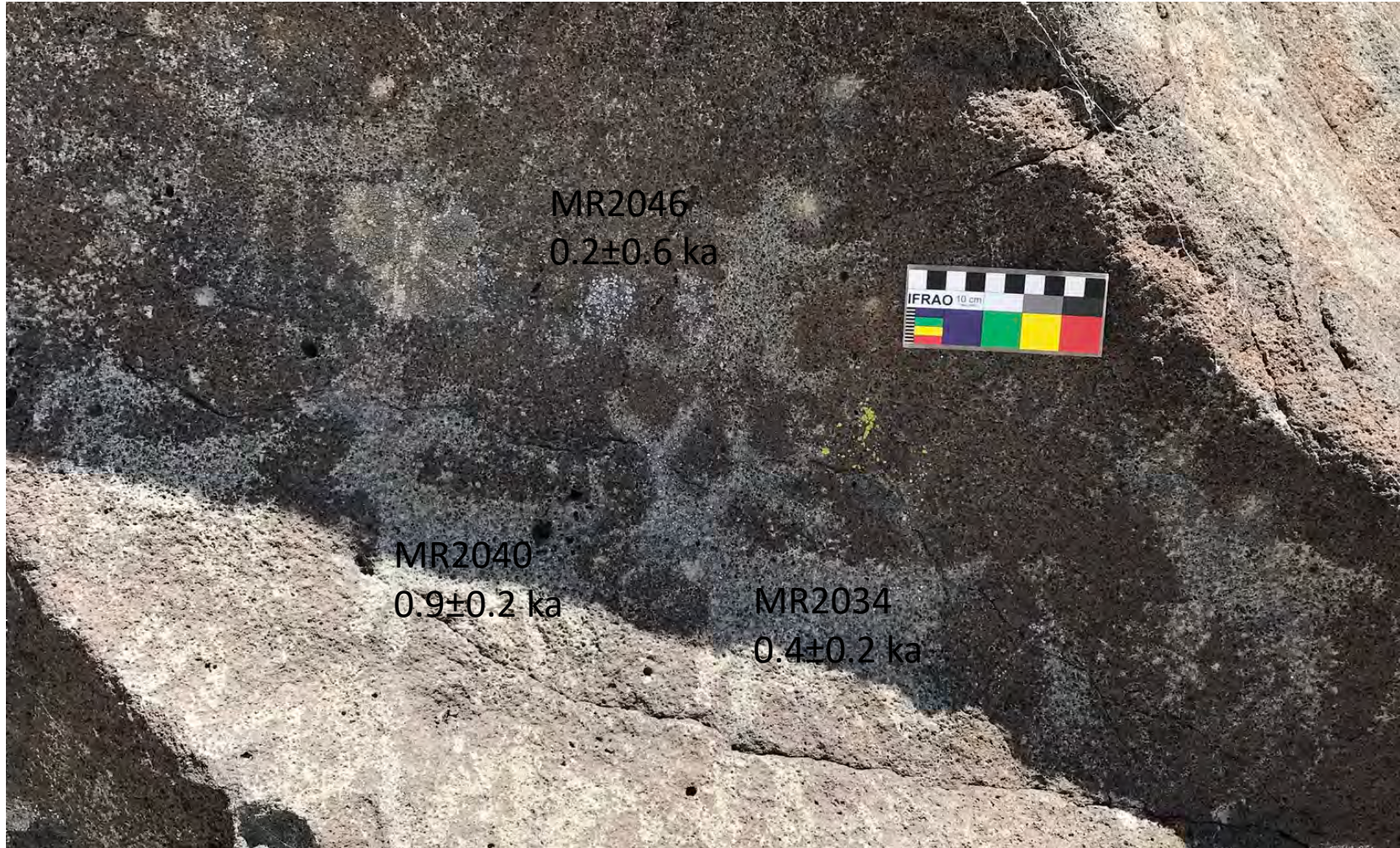

MR 2053

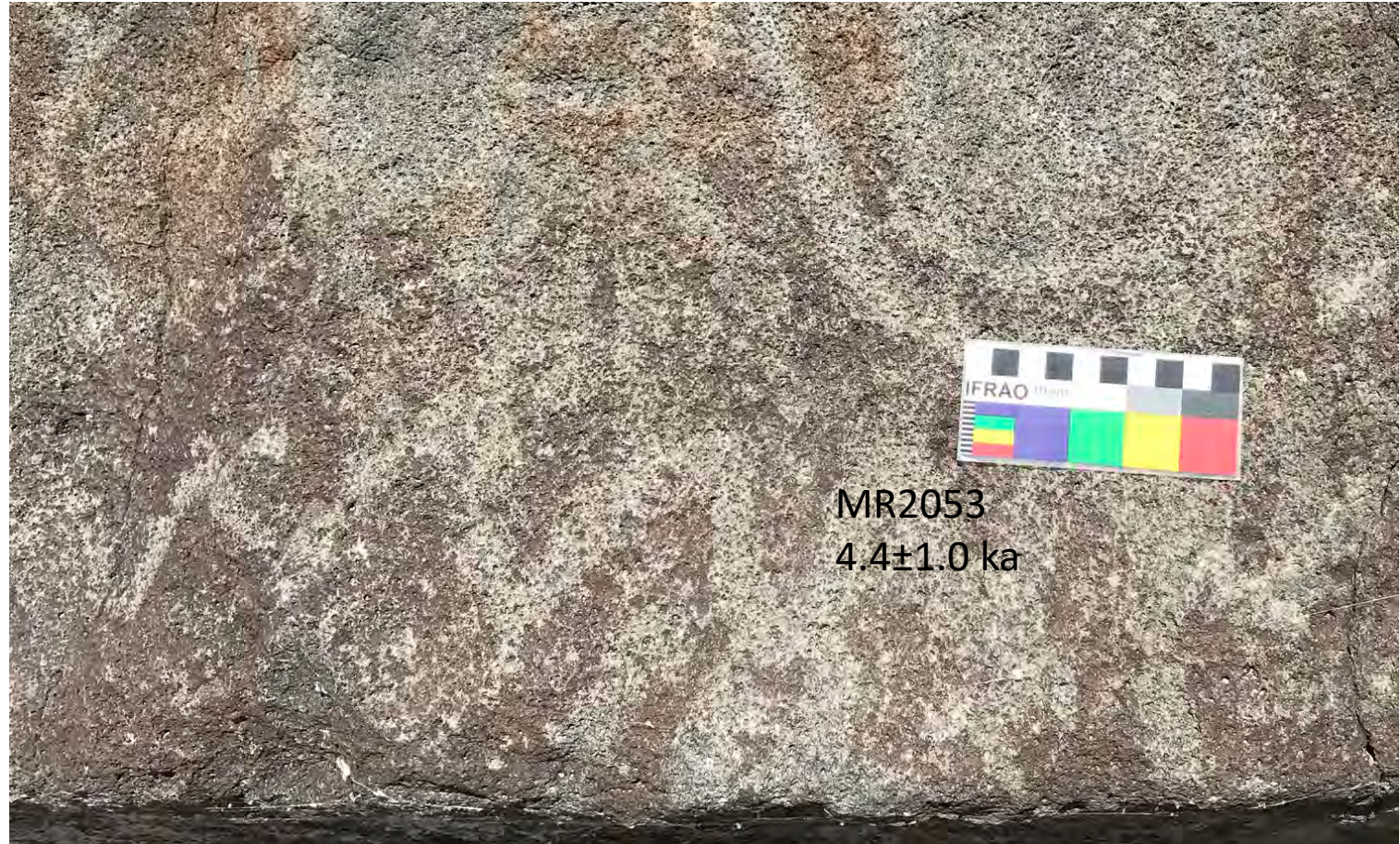

MR2053  
4.4±1.0 ka

MR 2059

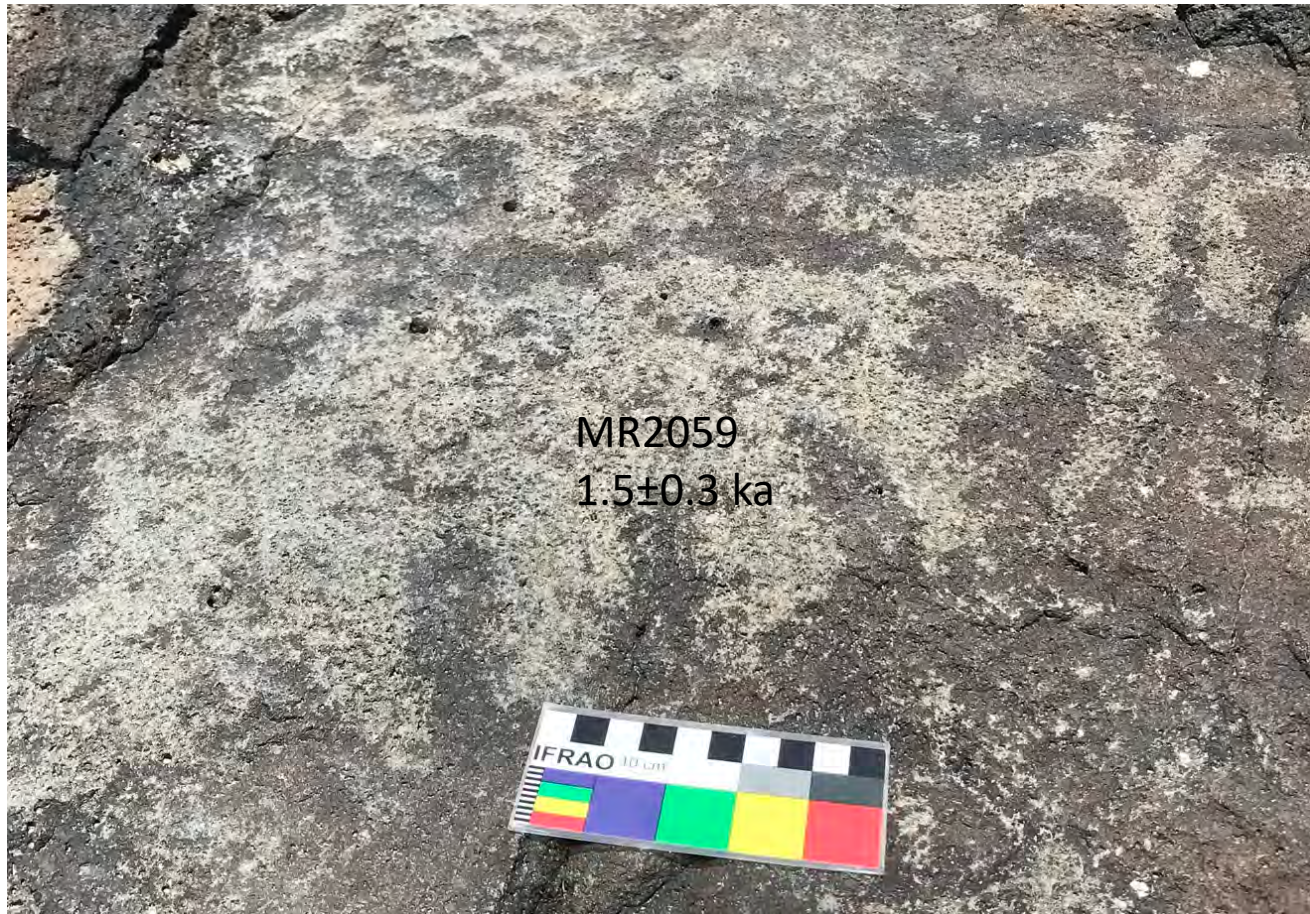

MR2066

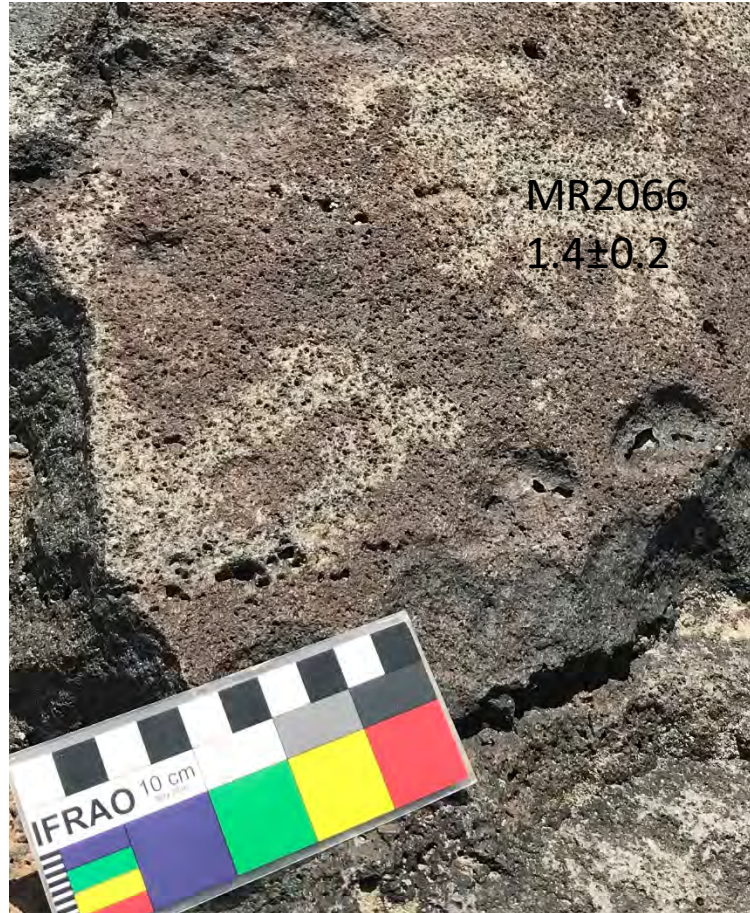

MR 2071

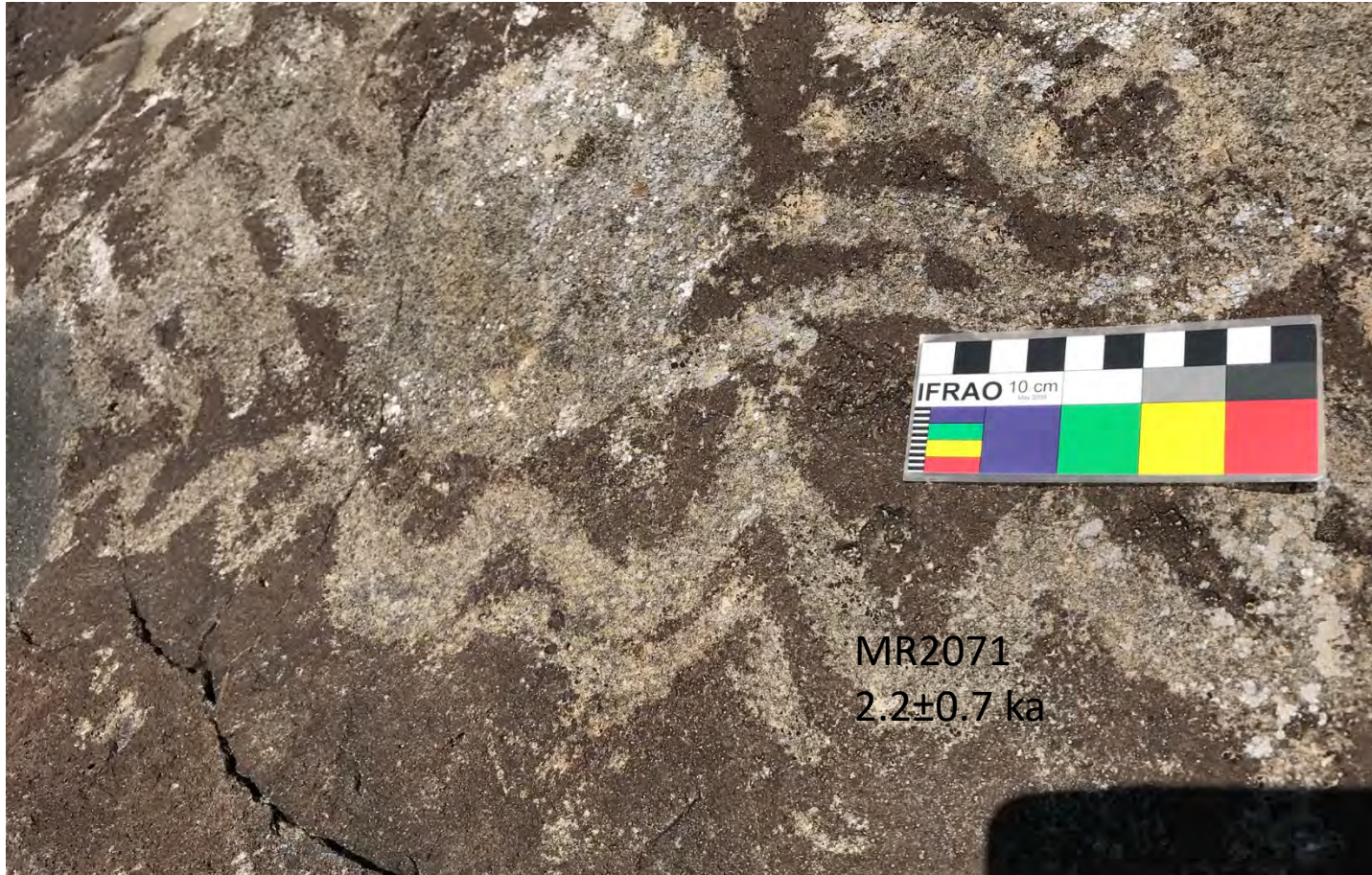

MR2071

2.2±0.7 ka

MR 2078

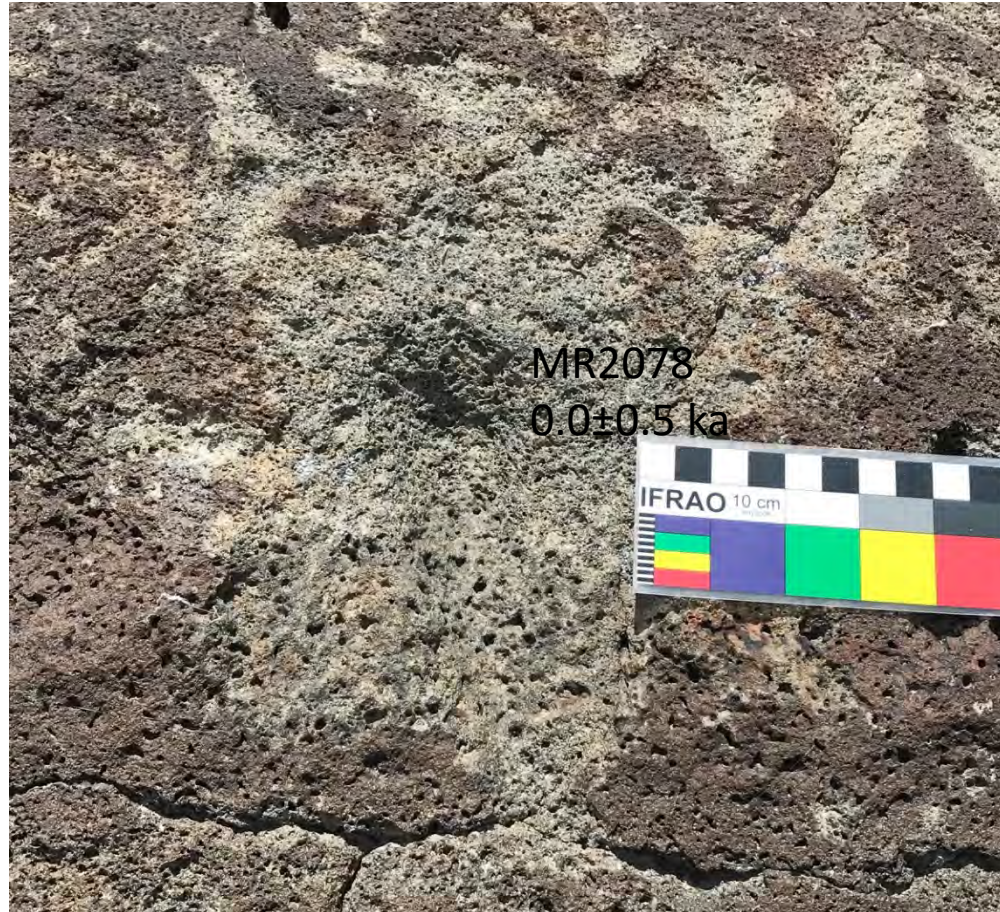

MR 2084

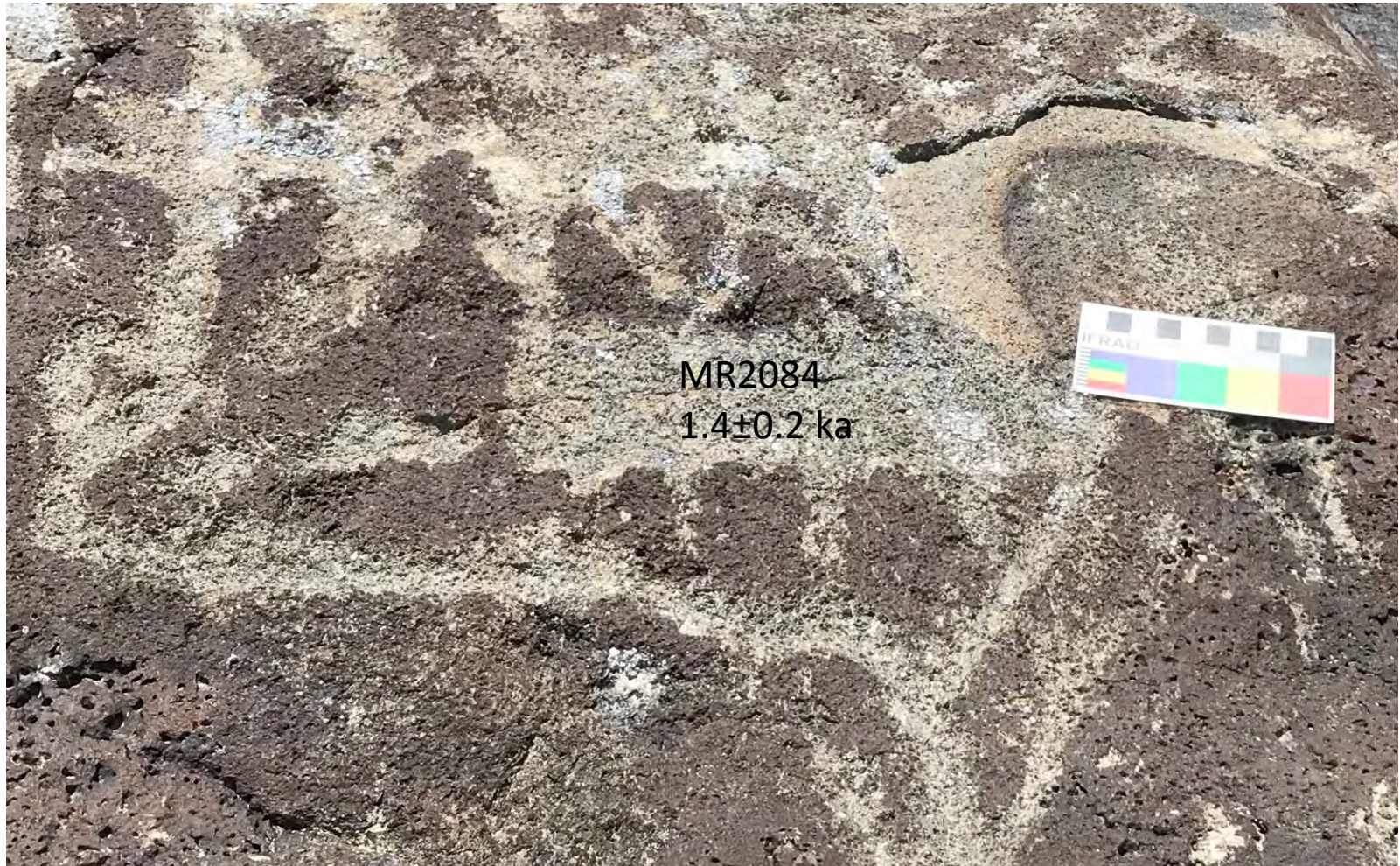

Supplement: S2 File — (PDF) [file pone.0263189.s003.pdf]

LR 2307

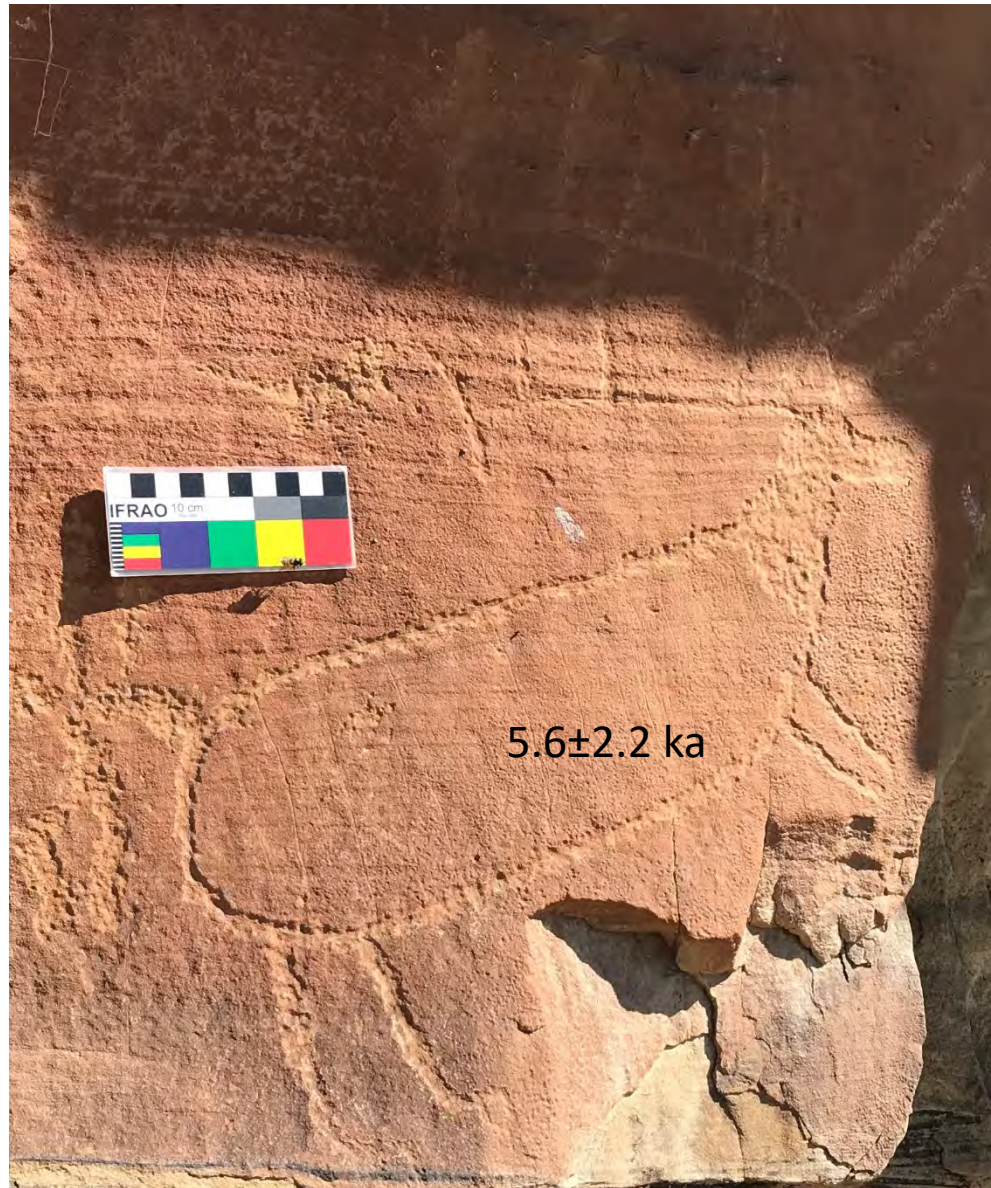

LR2315

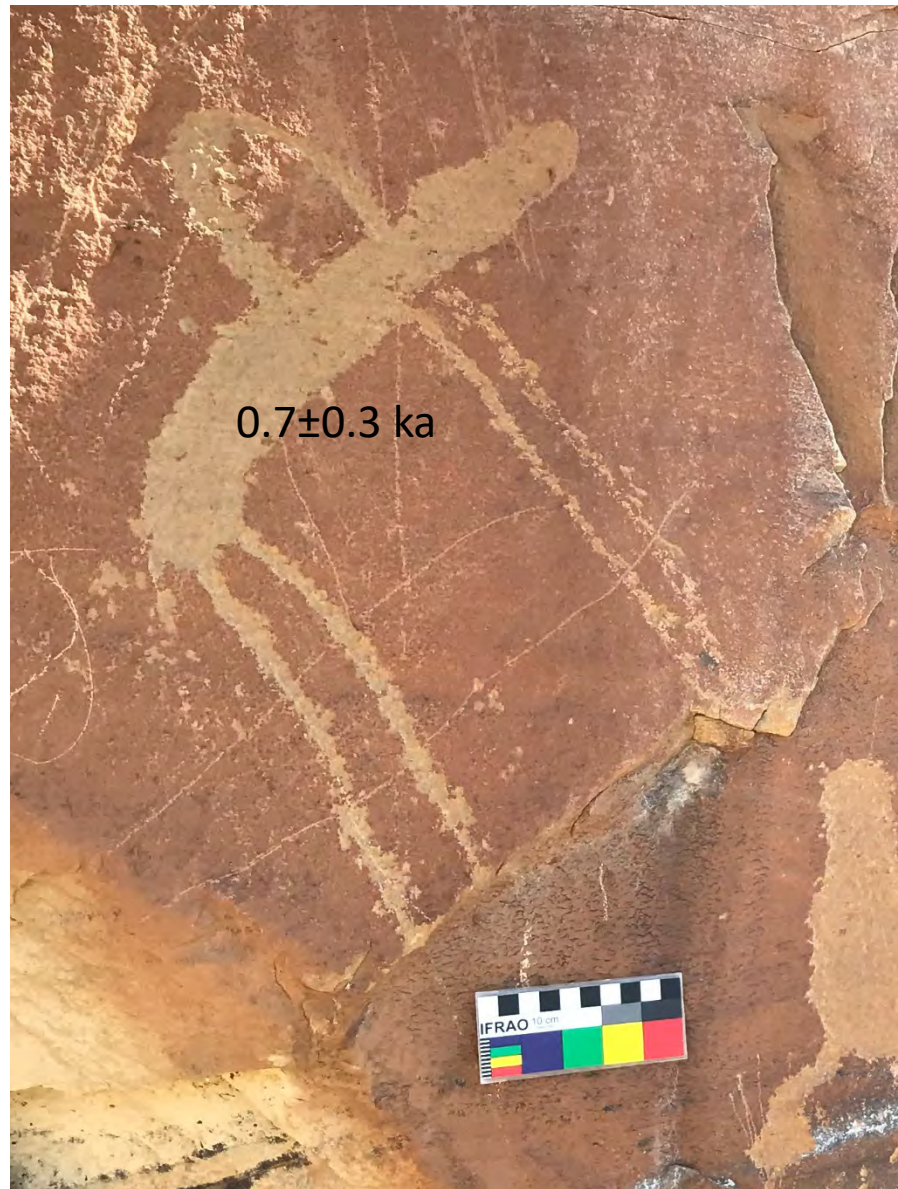

LR2327

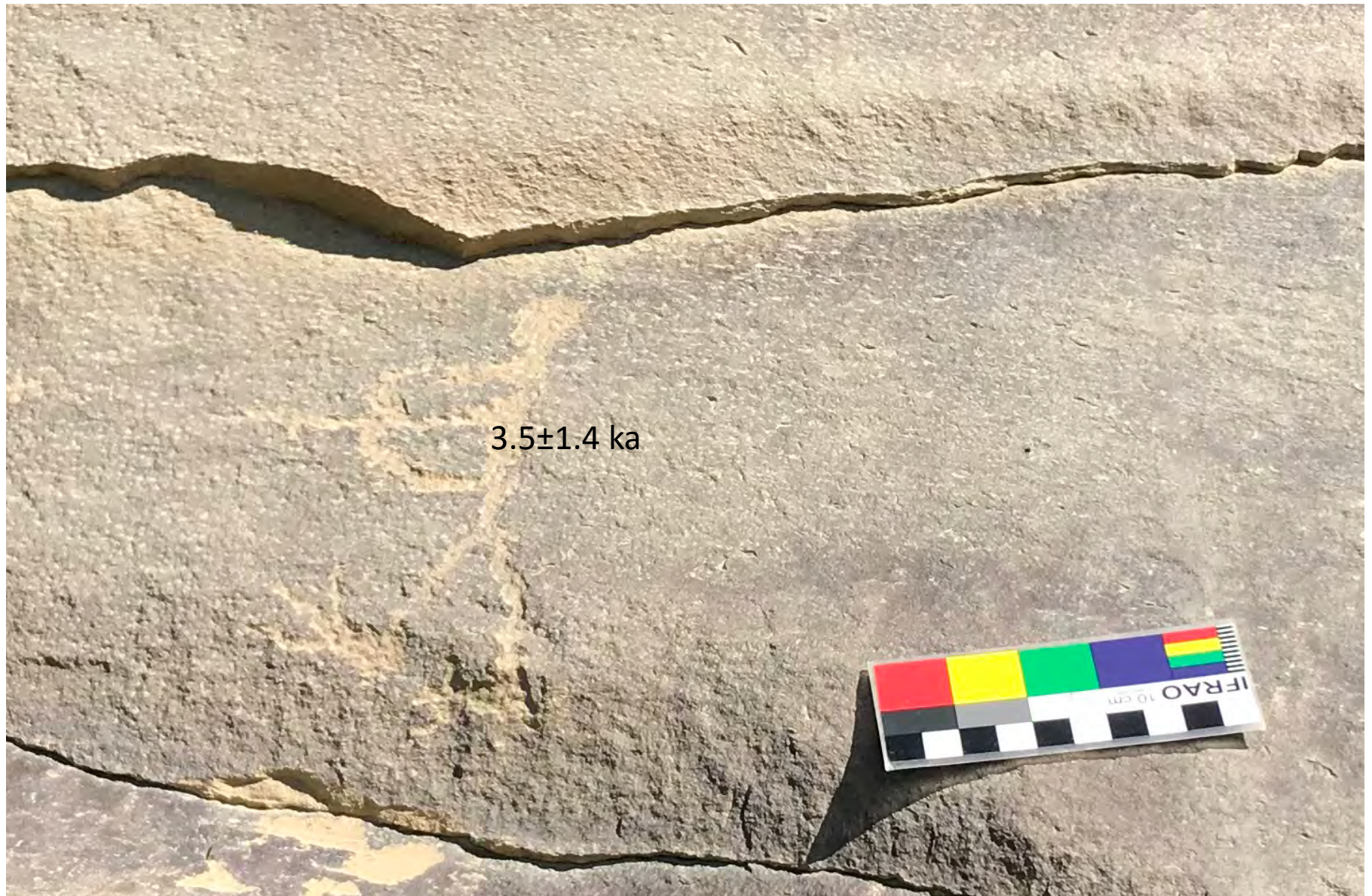

LR2335

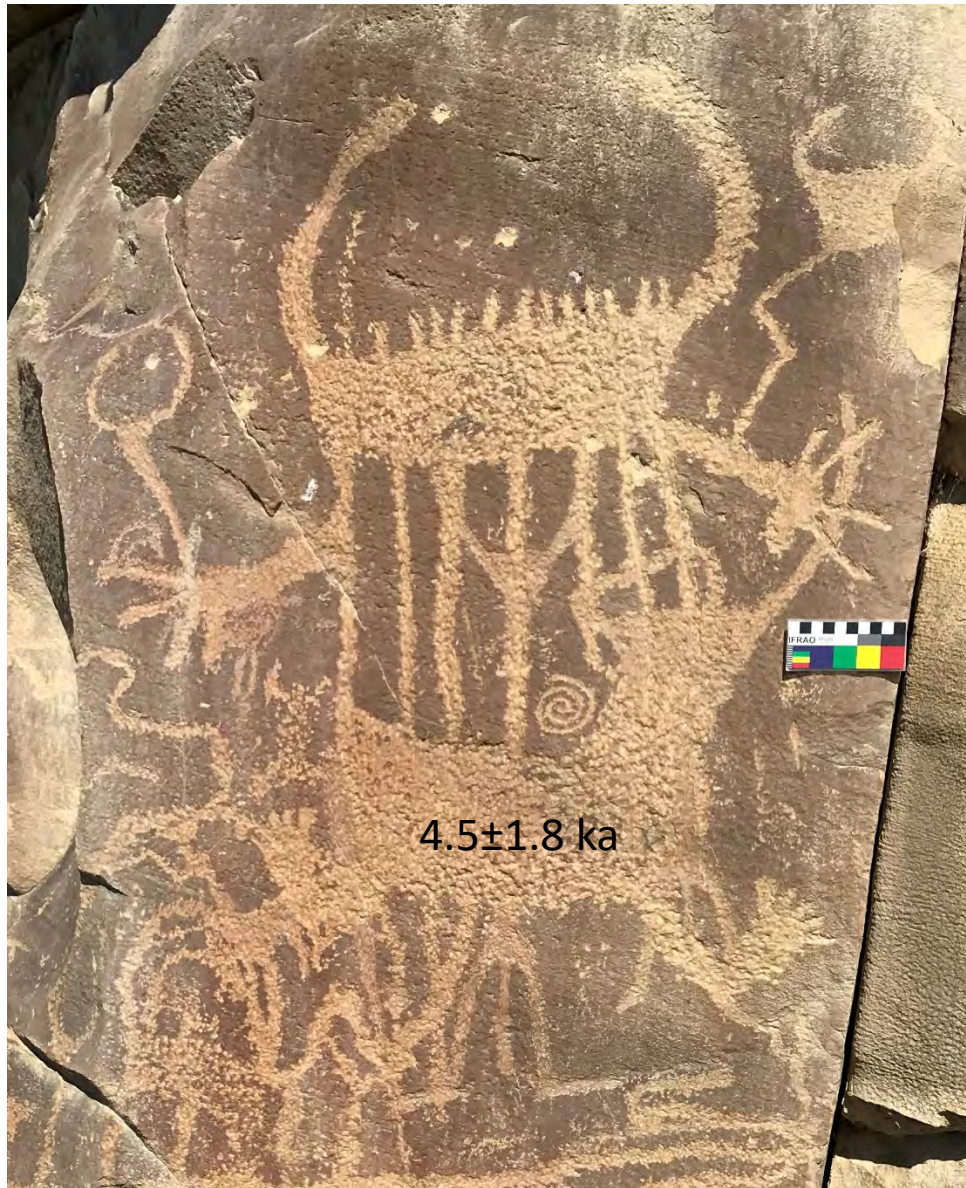

$4.5 \pm 1.8$  ka

# Panel 48

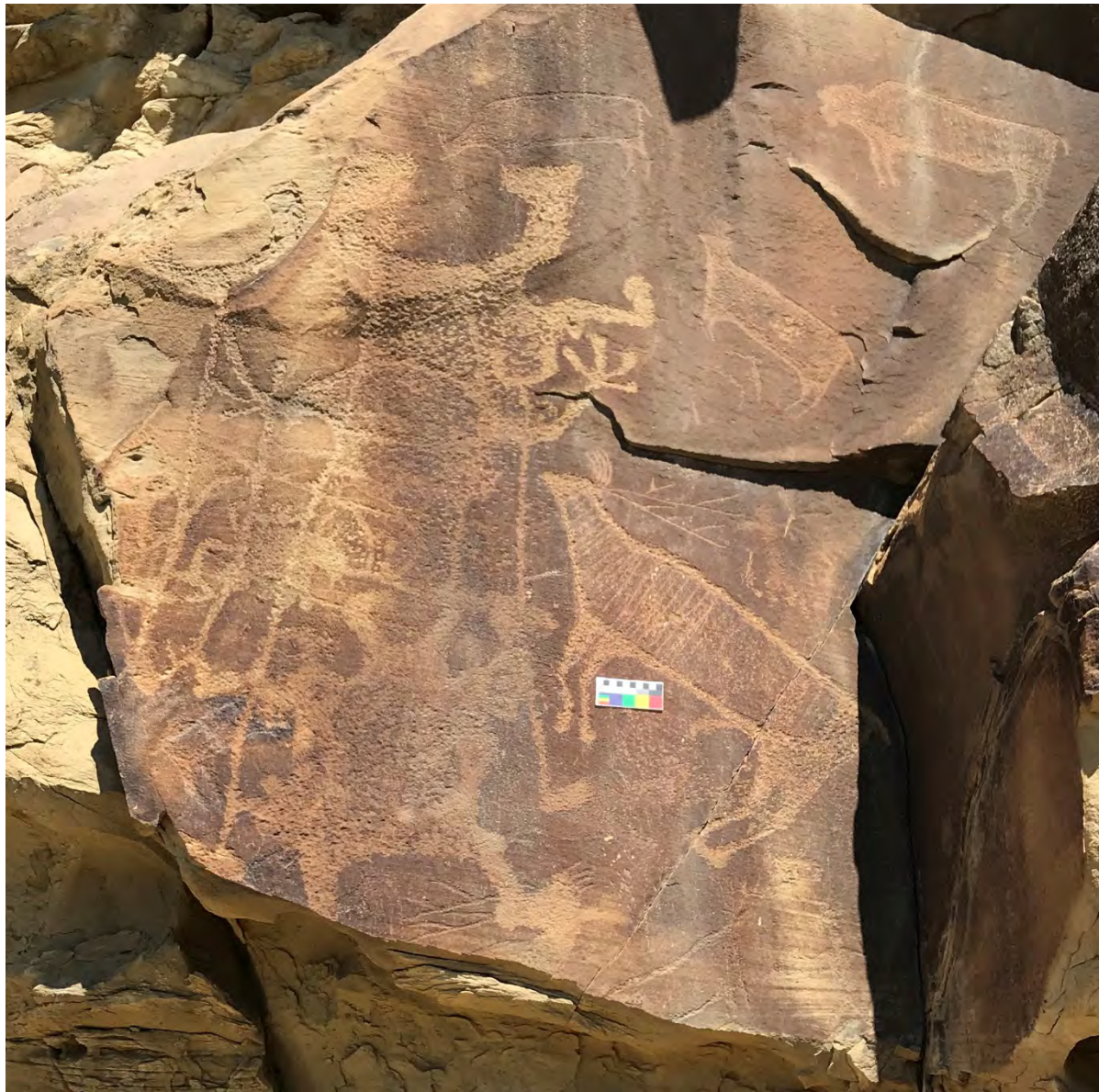

LR2345

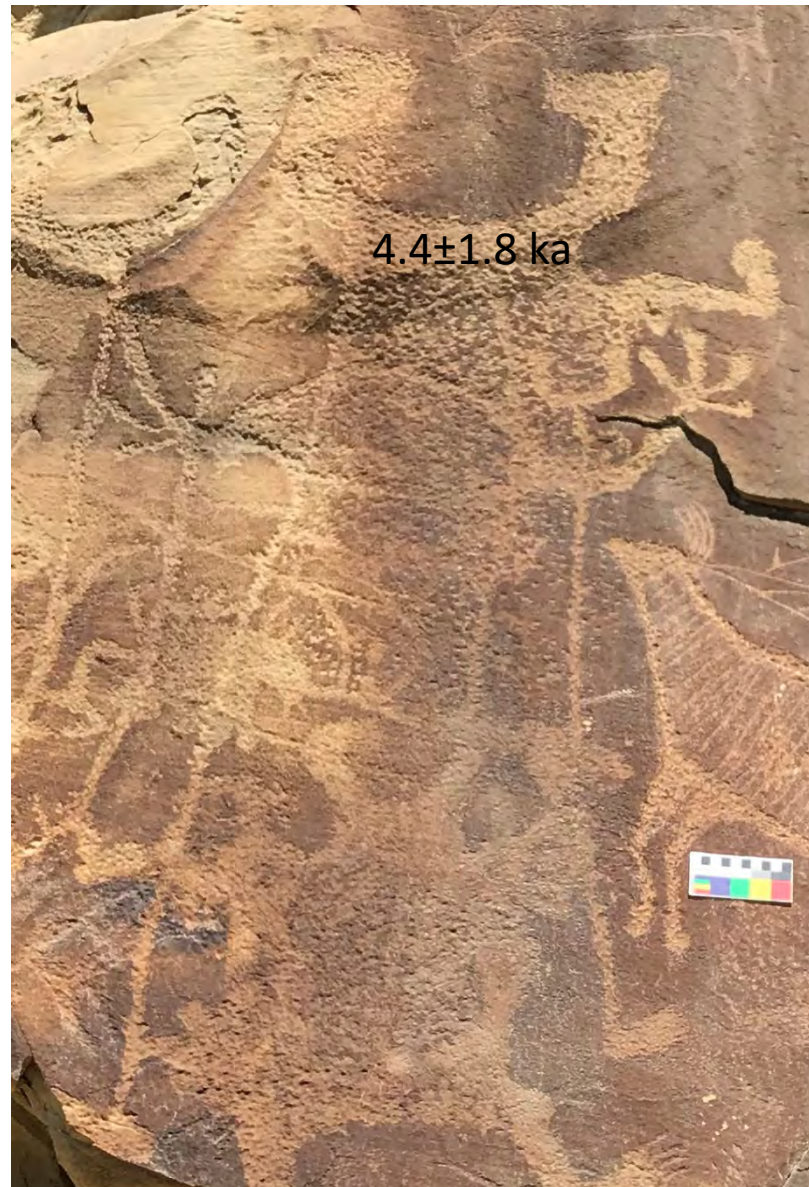

LR2351/63/76

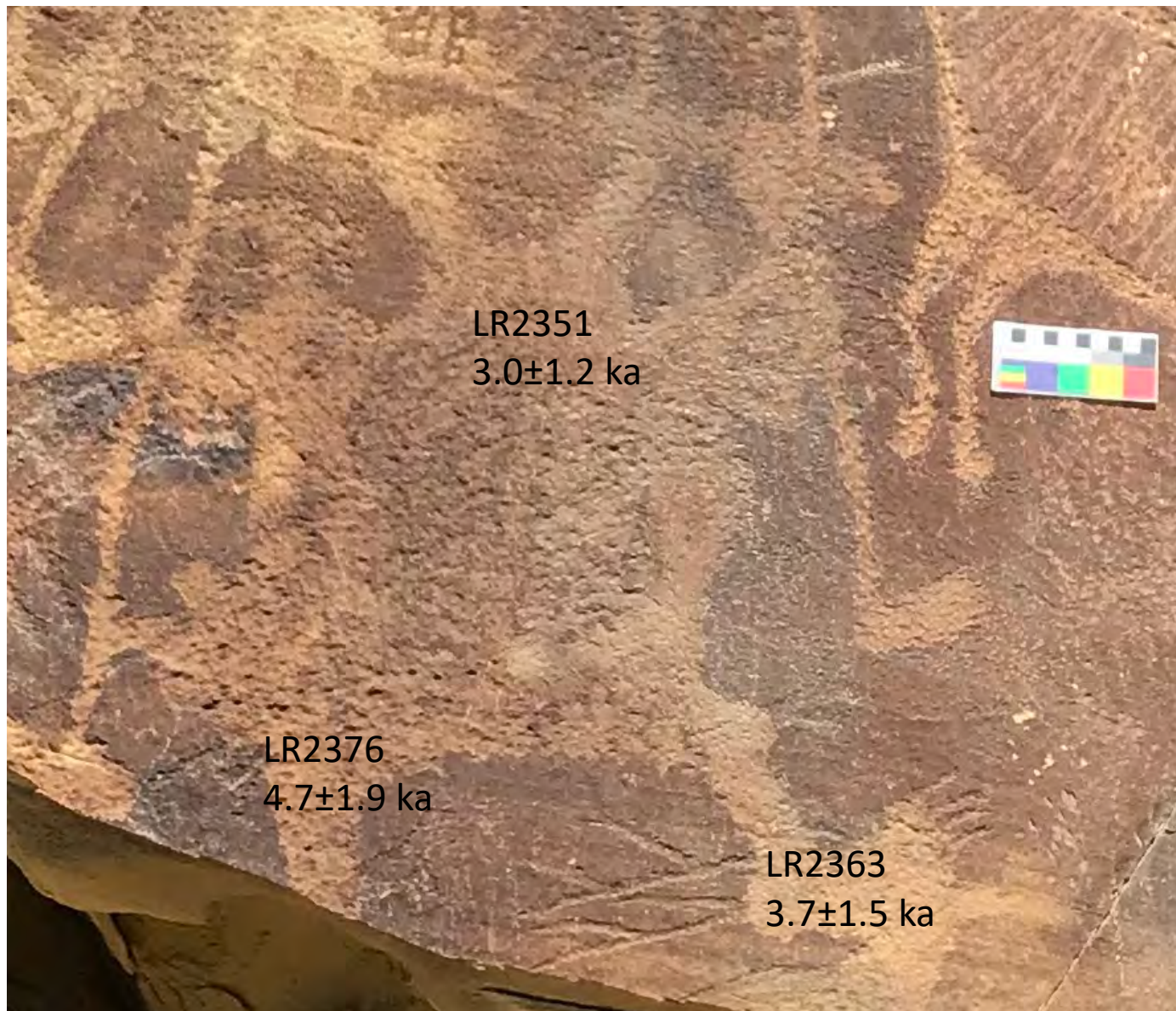

LR2357

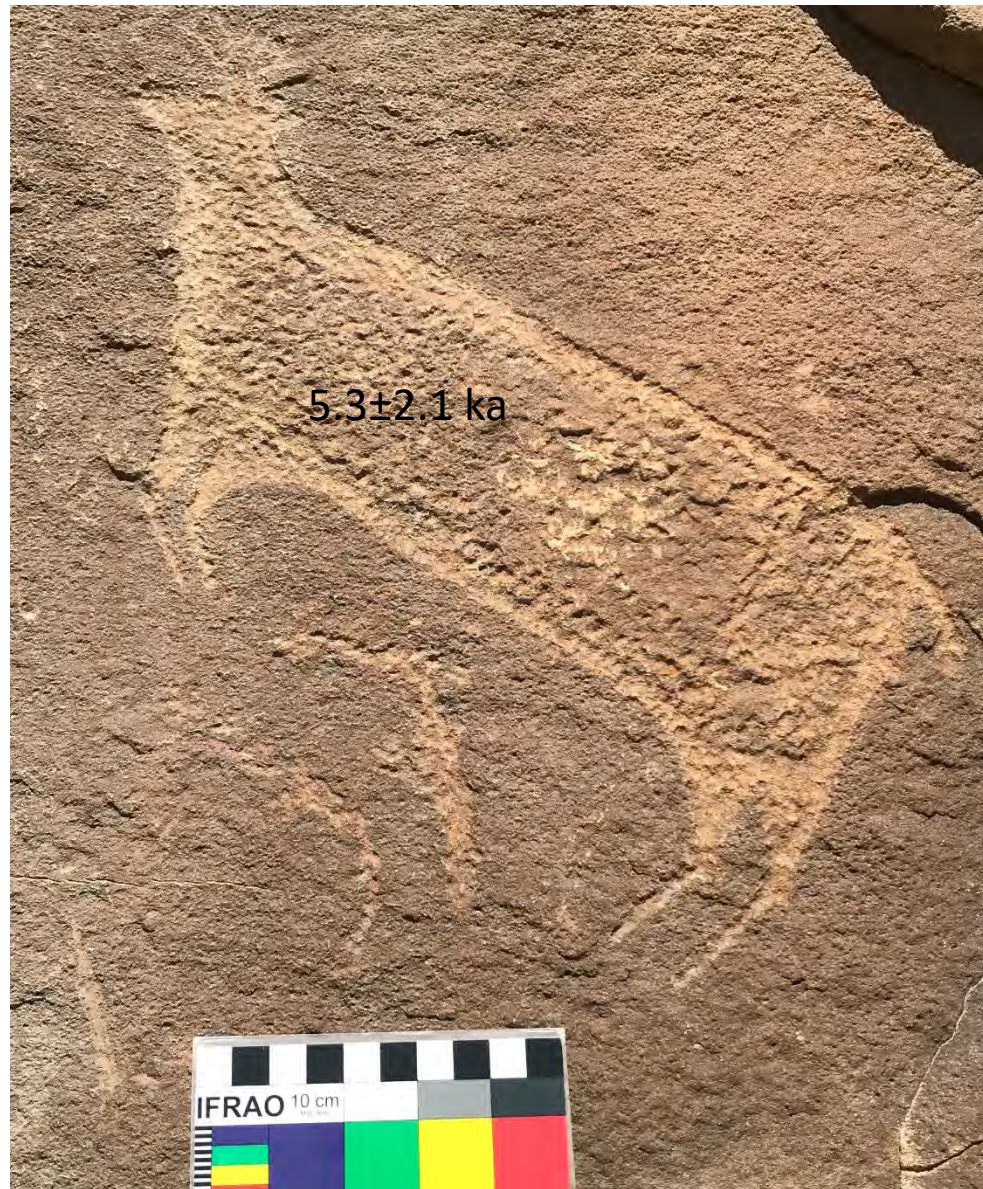

LR2369

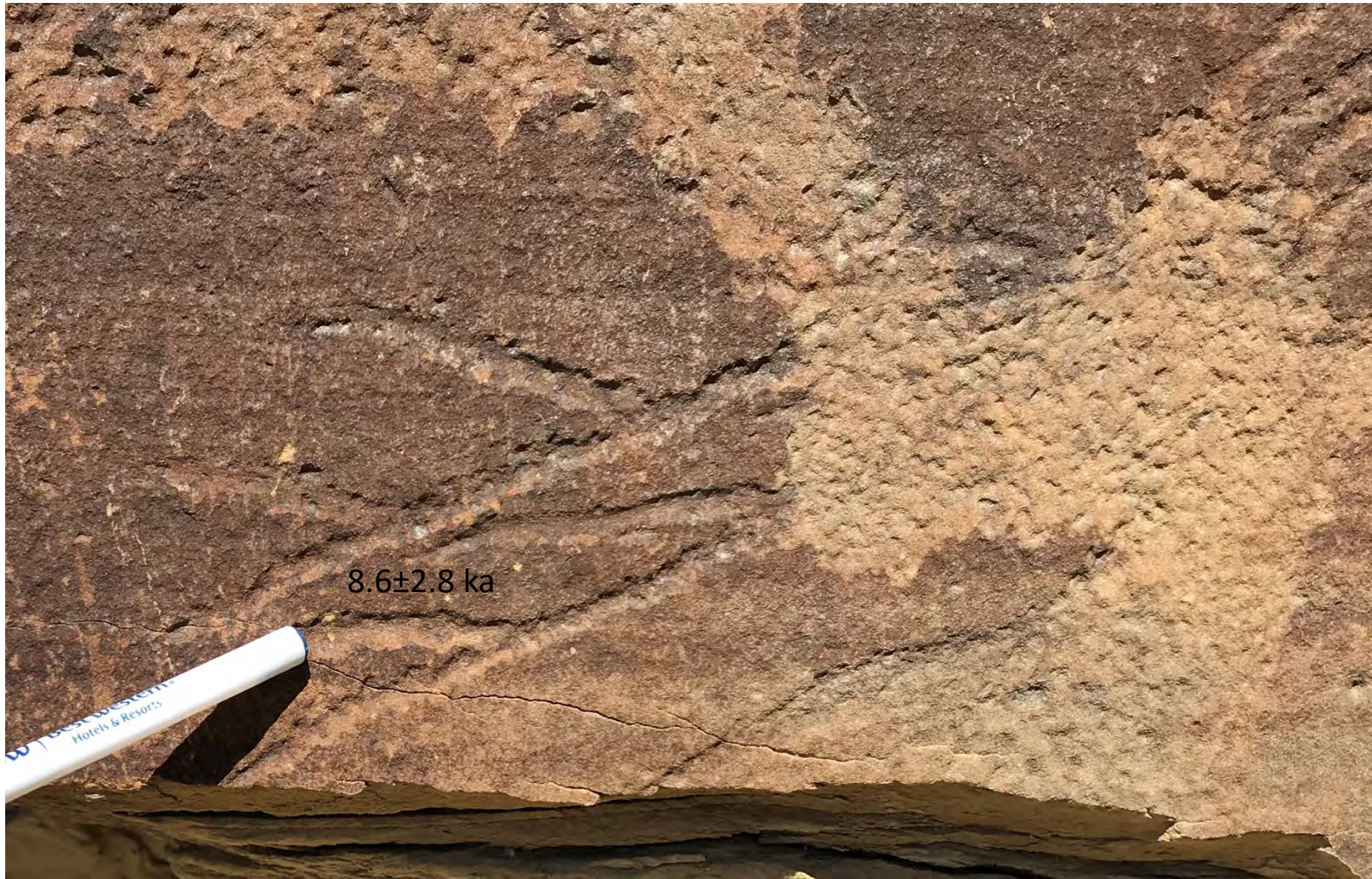

8.6±2.8 ka

LR2382

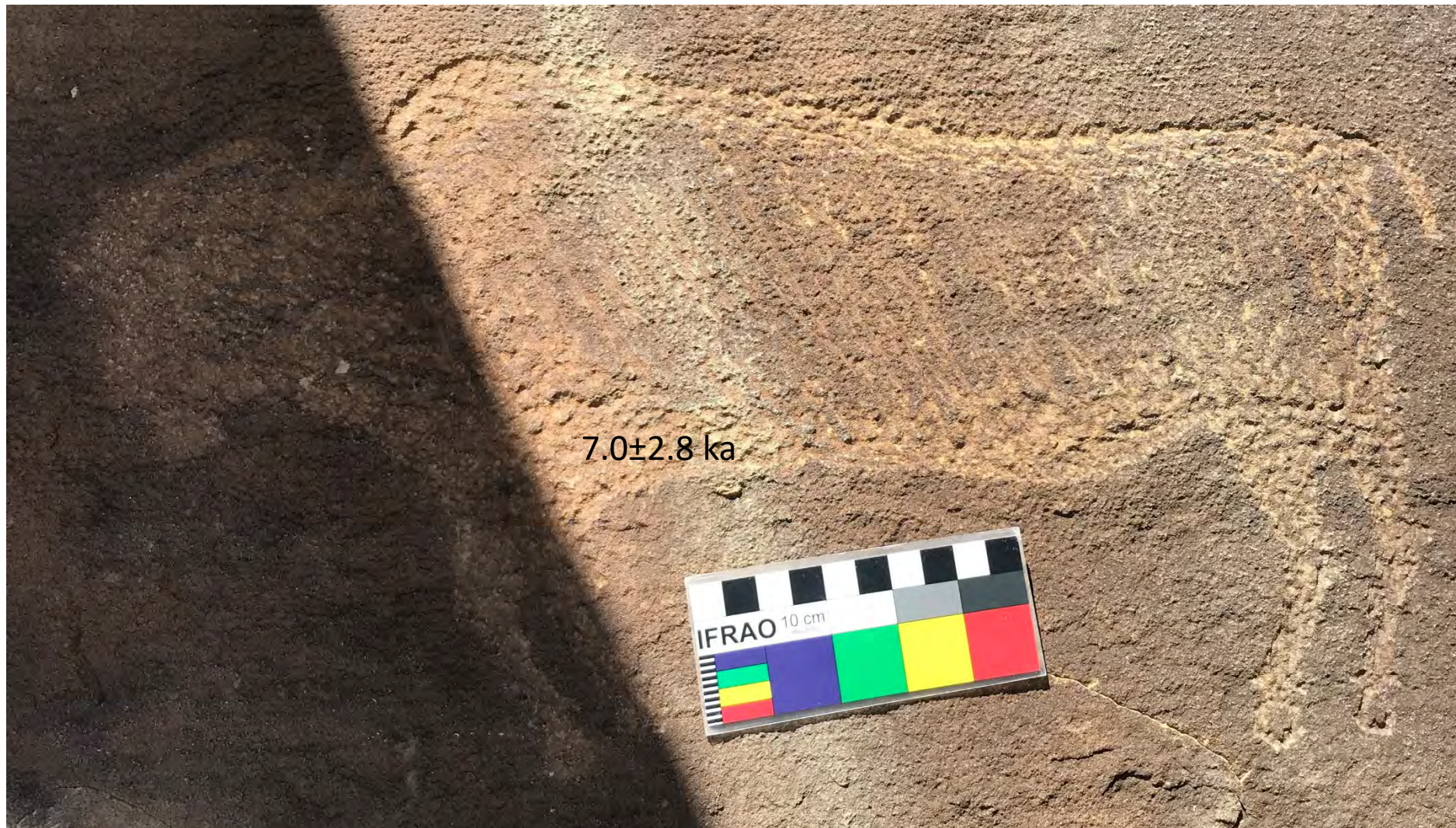

LR2390

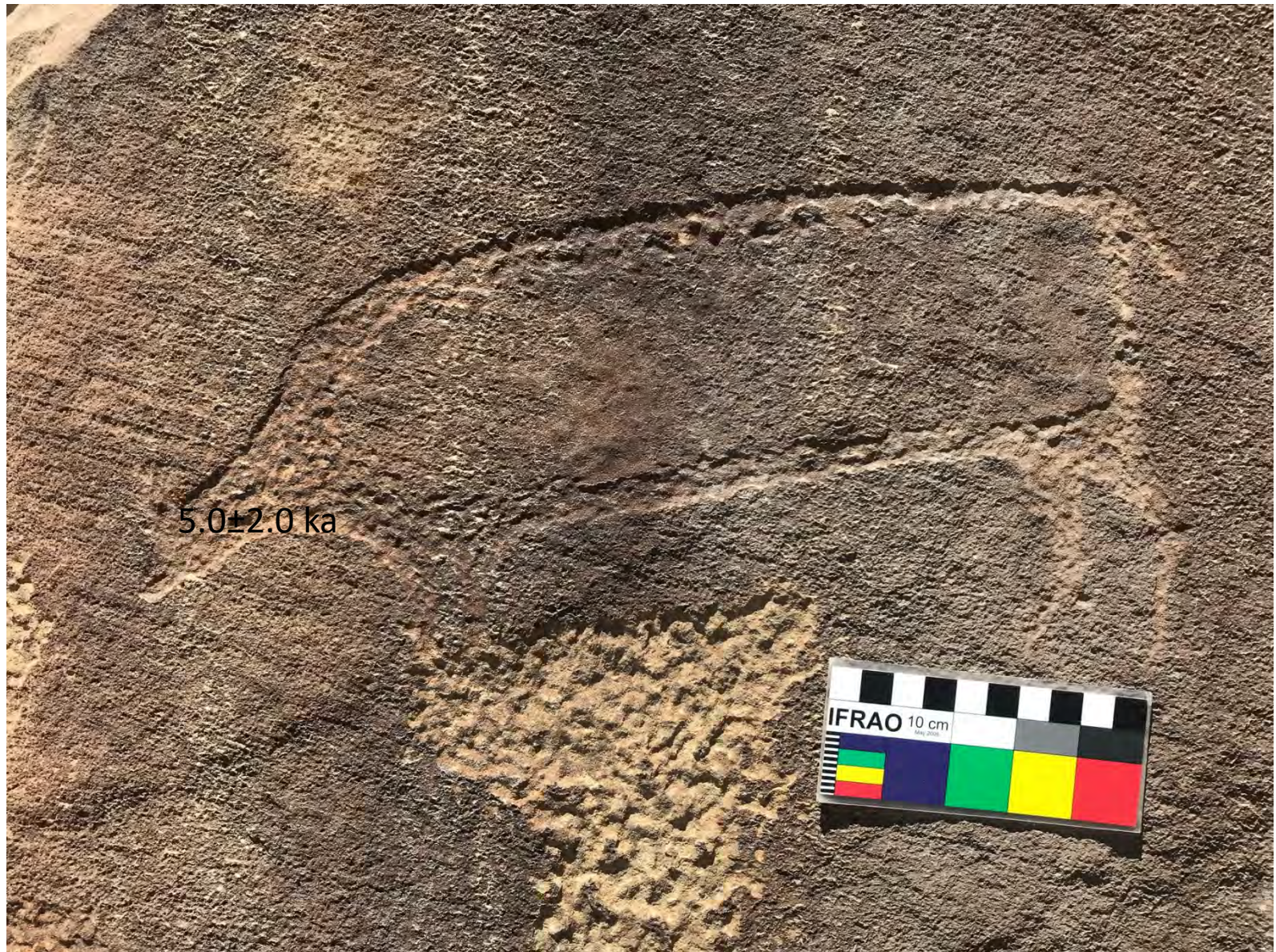

LR2400

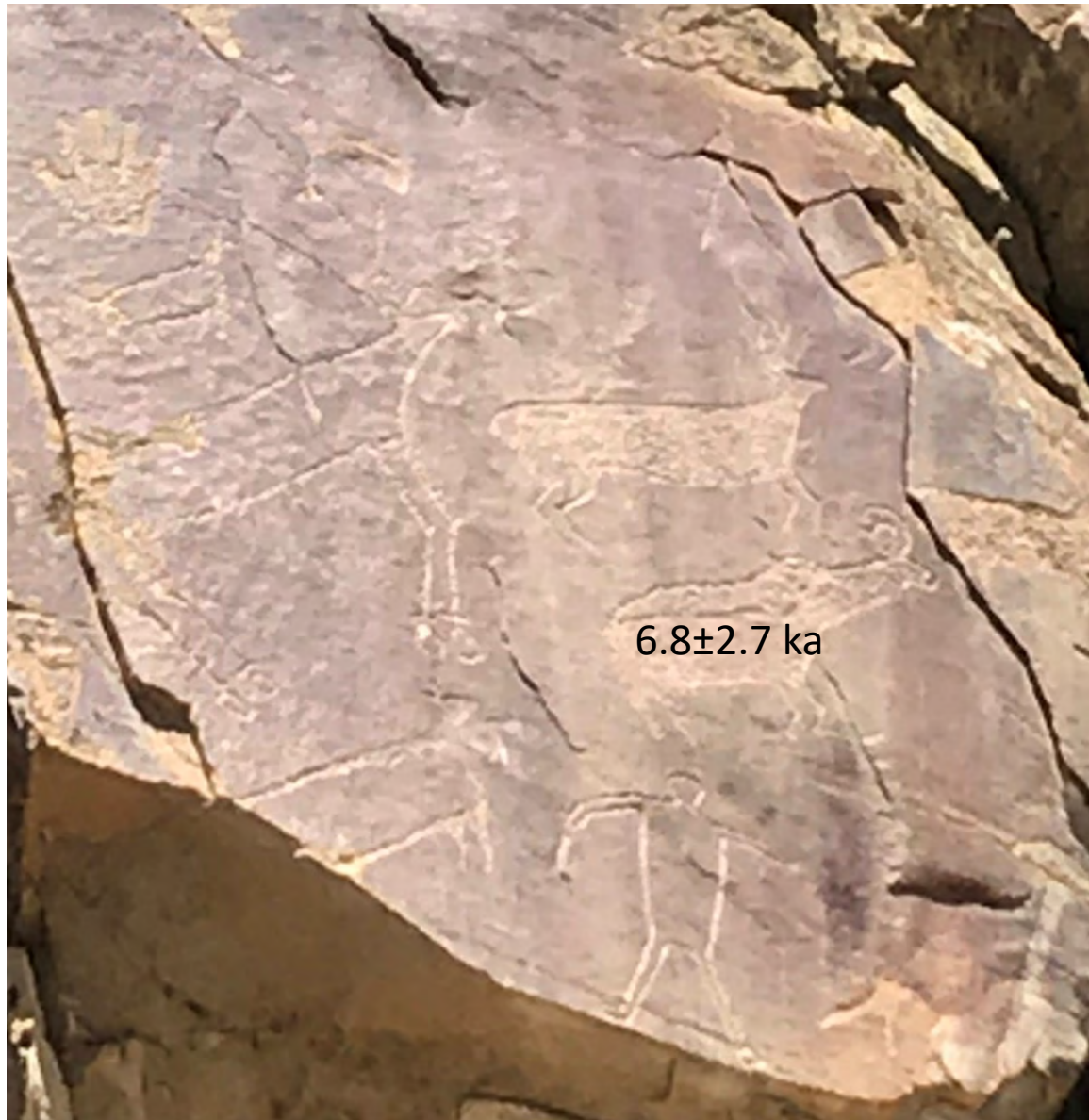

LR2407

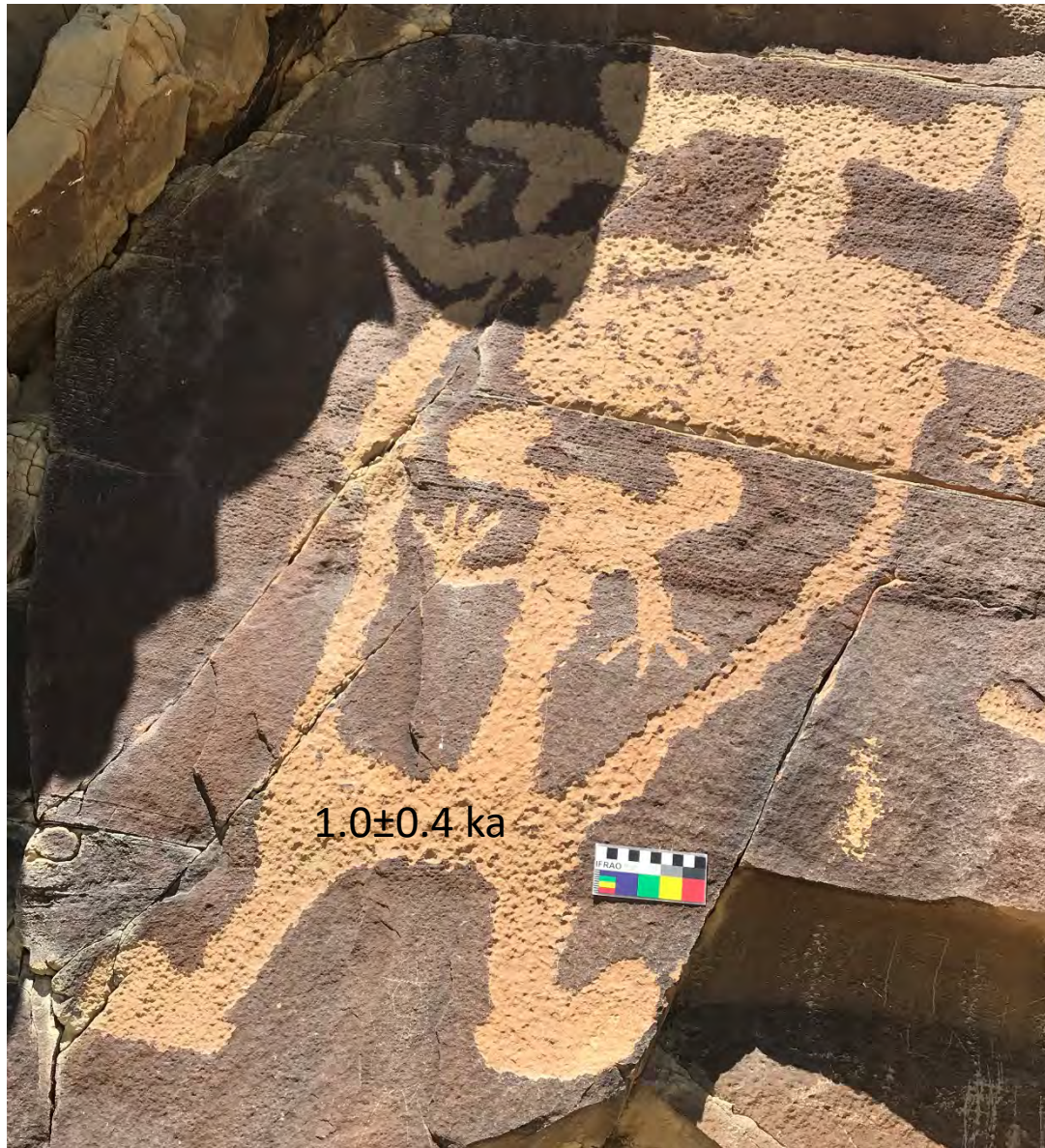

1.0±0.4 ka

LR2411

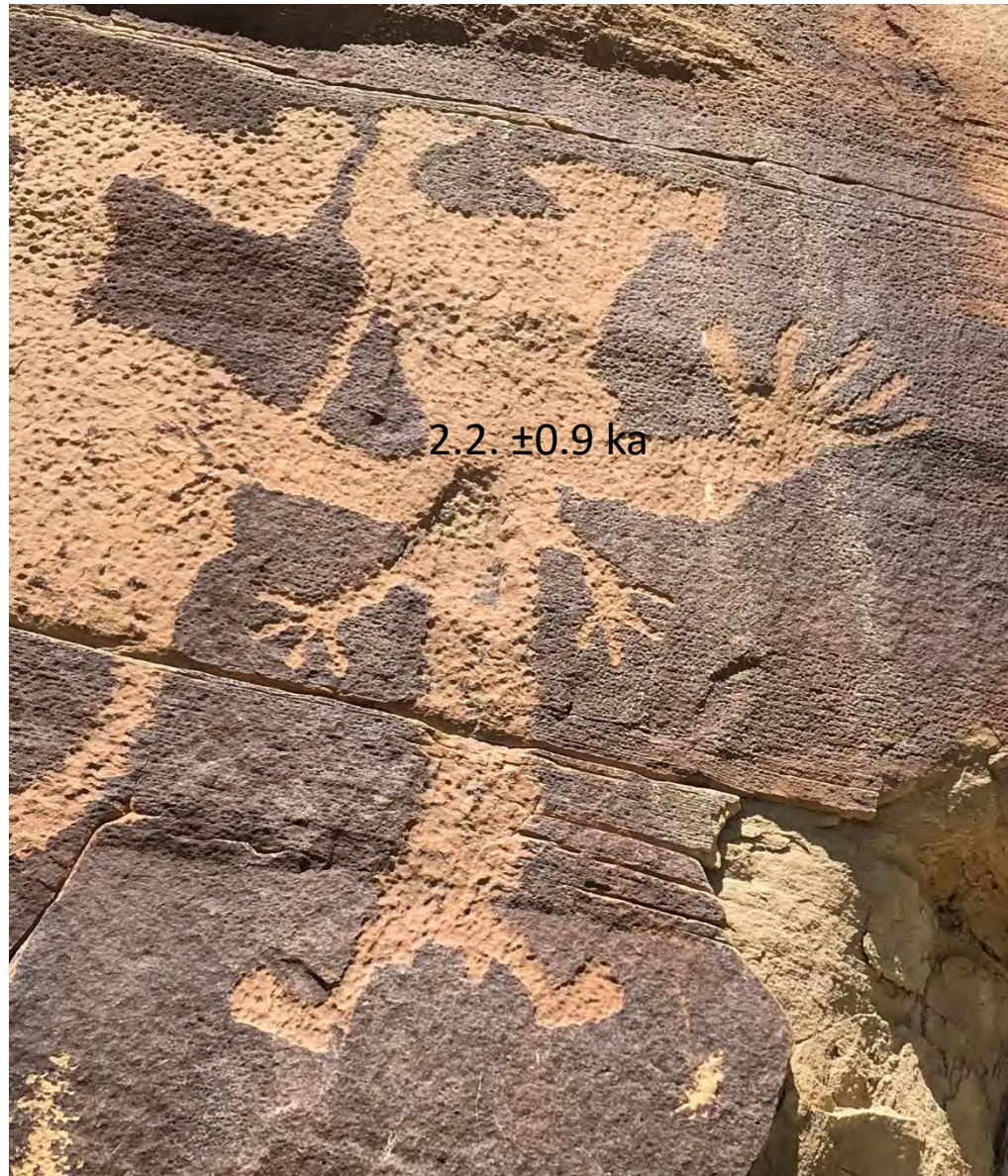

LR2417

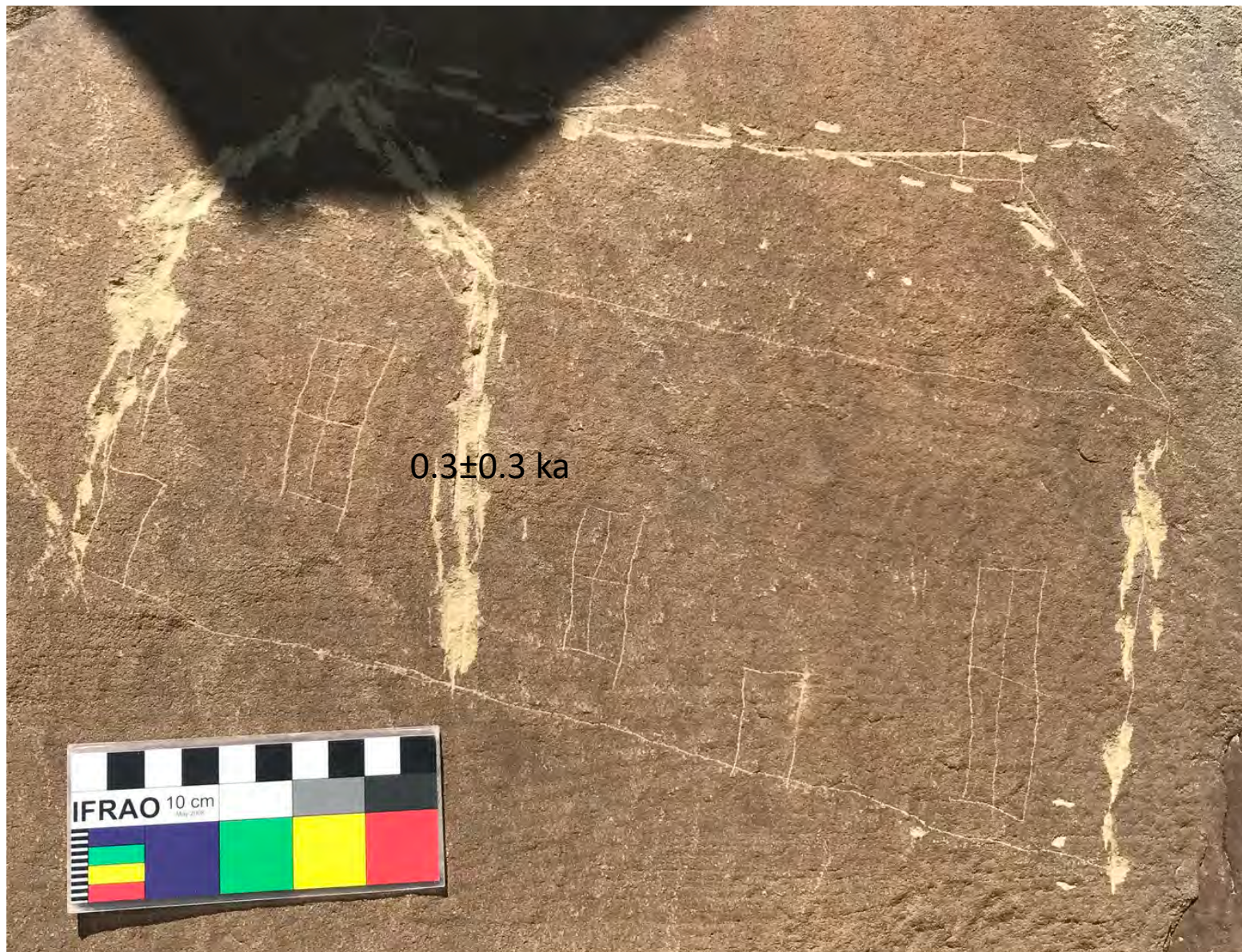

LR2419

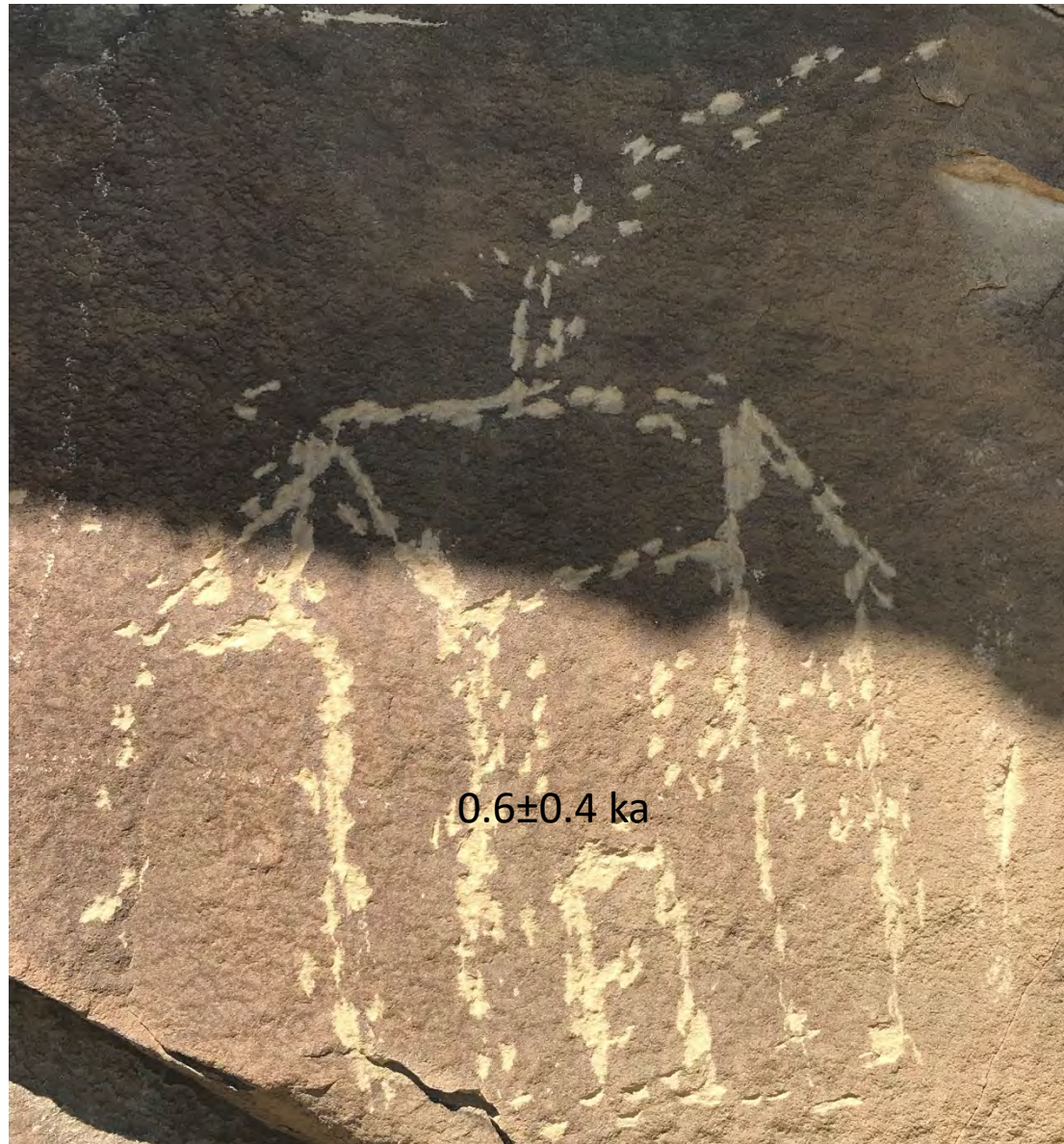

LR2426/30

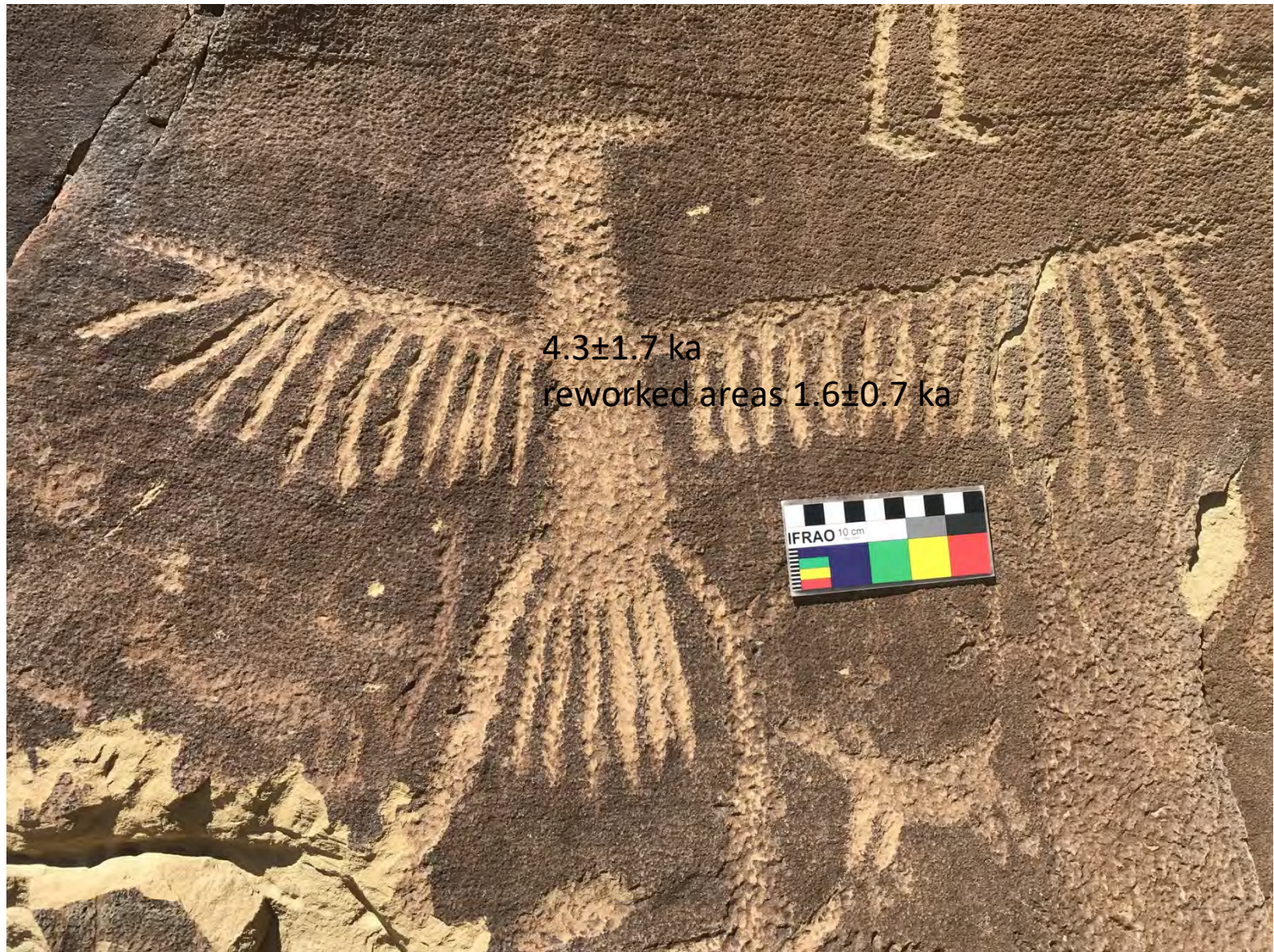

LR2437

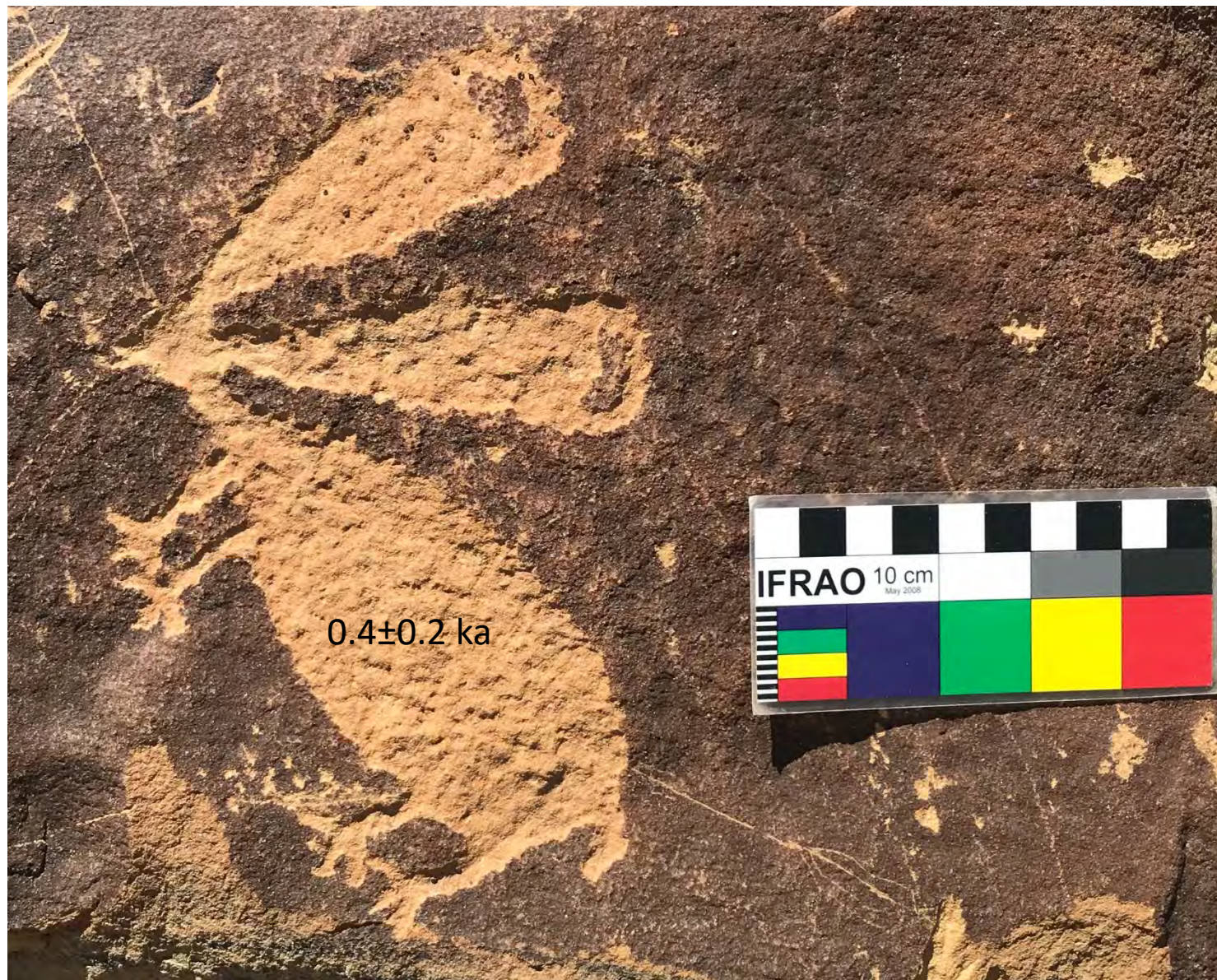

Supplement: S3 File — (PDF) [file pone.0263189.s004.pdf]

PC2447/55

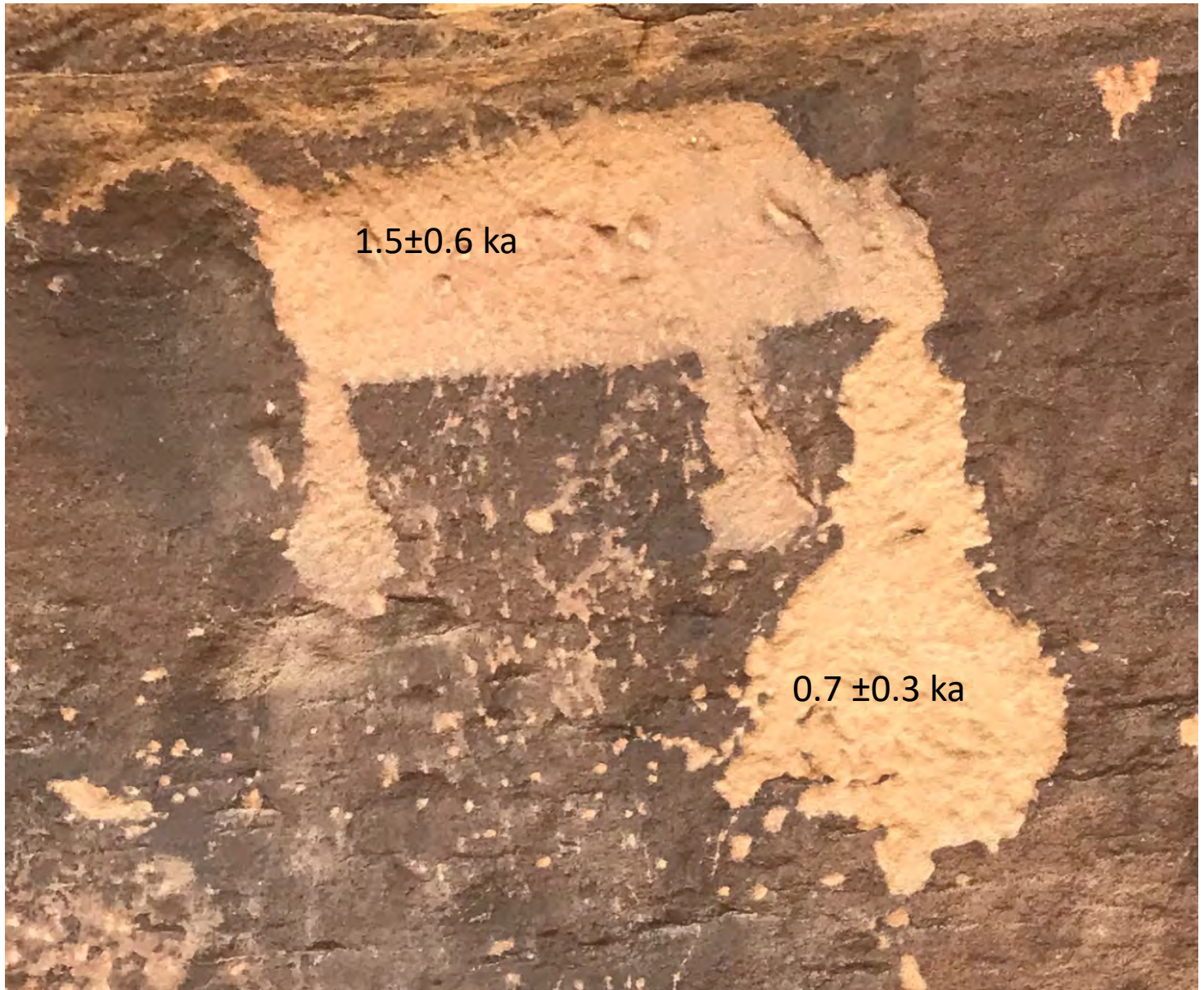

PC2458

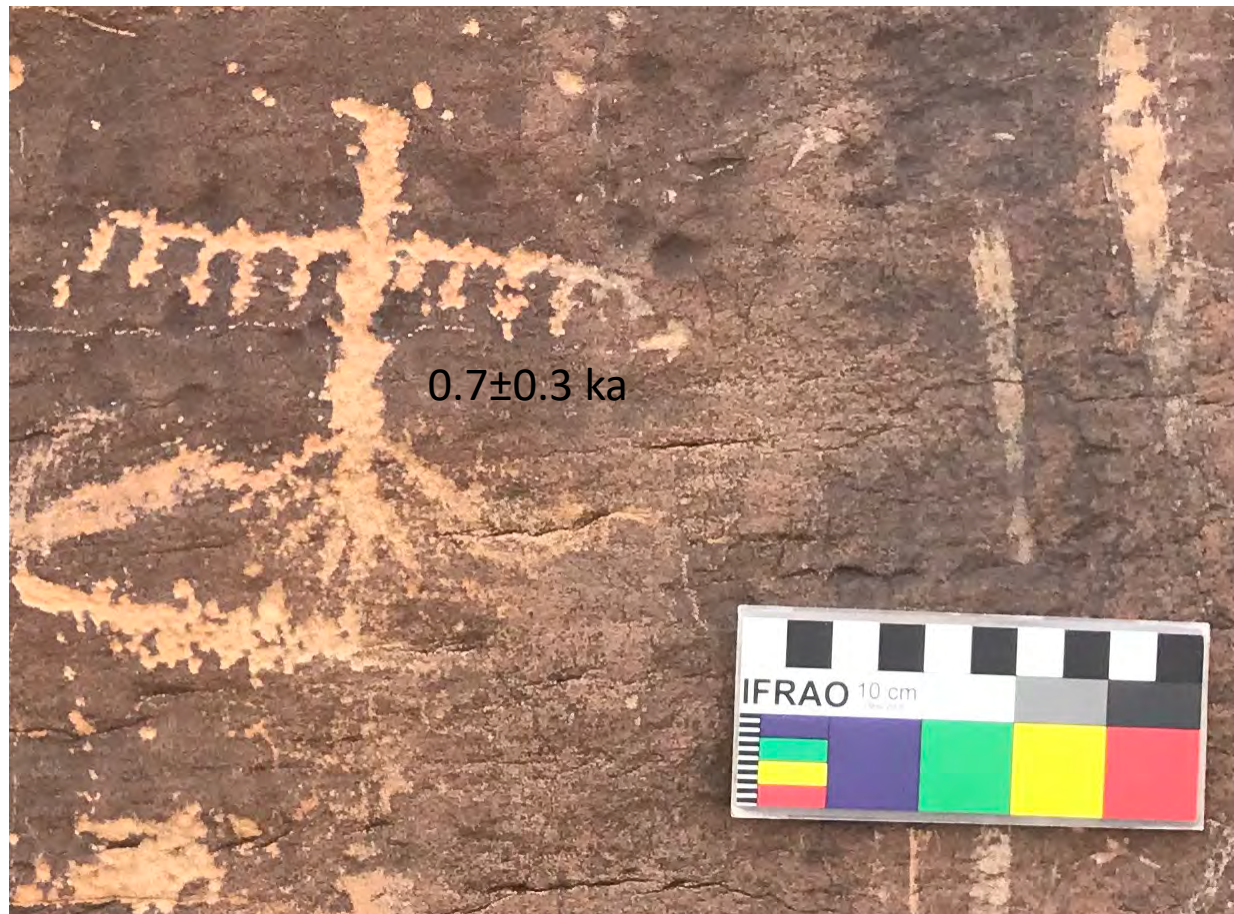

PC2465

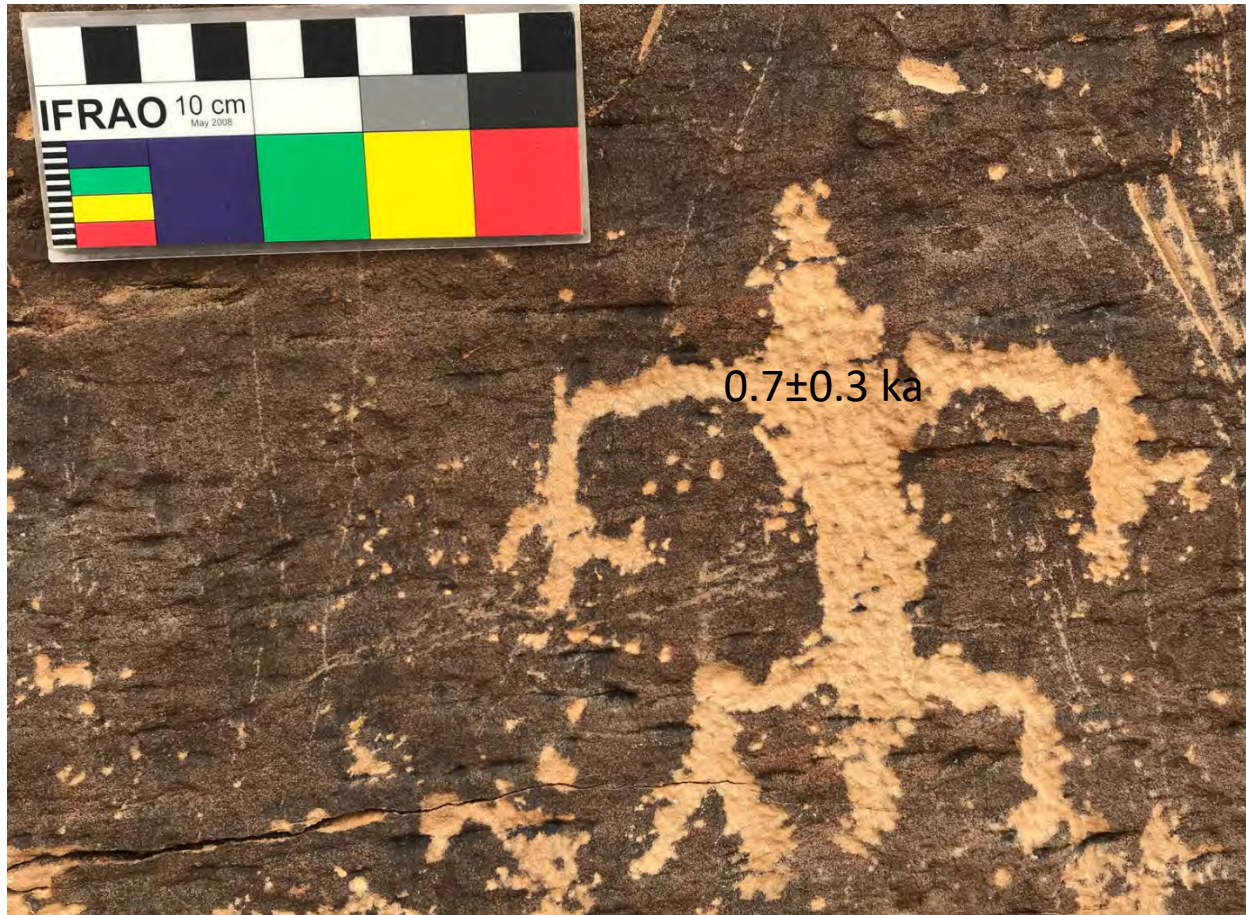

PC2472/80

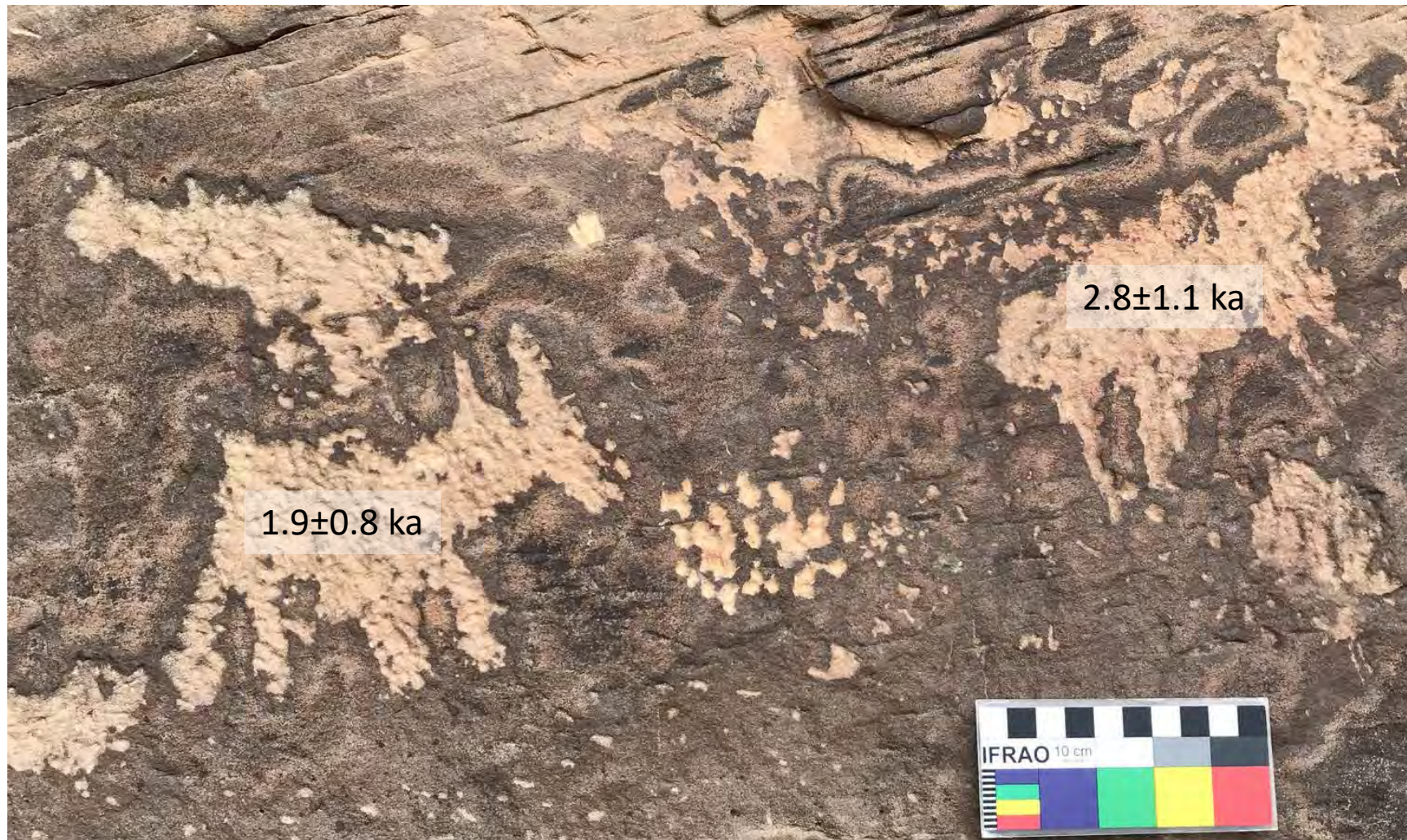

PC2485

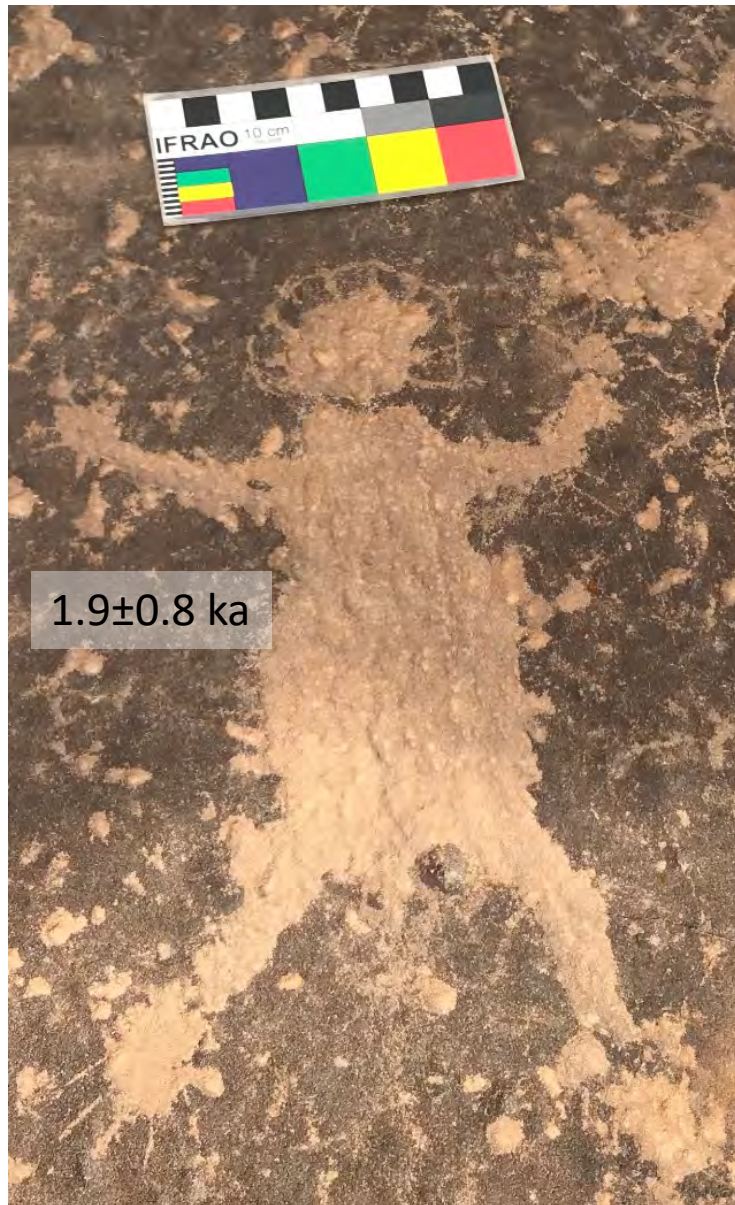

PC2493

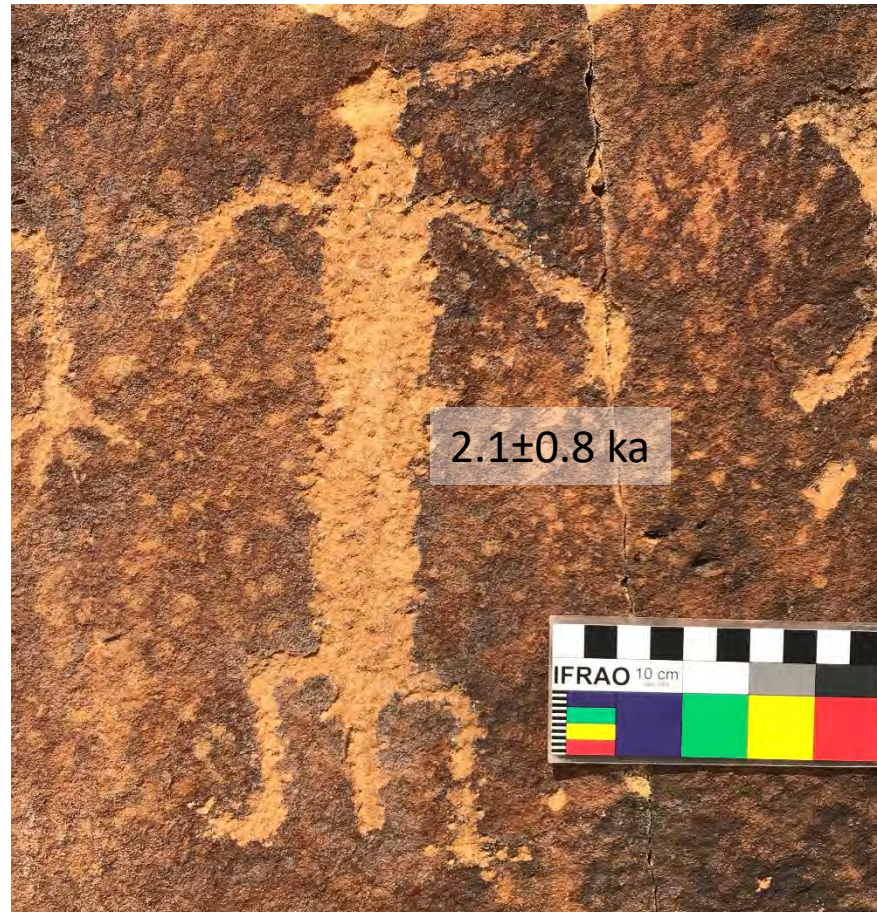

PC2501

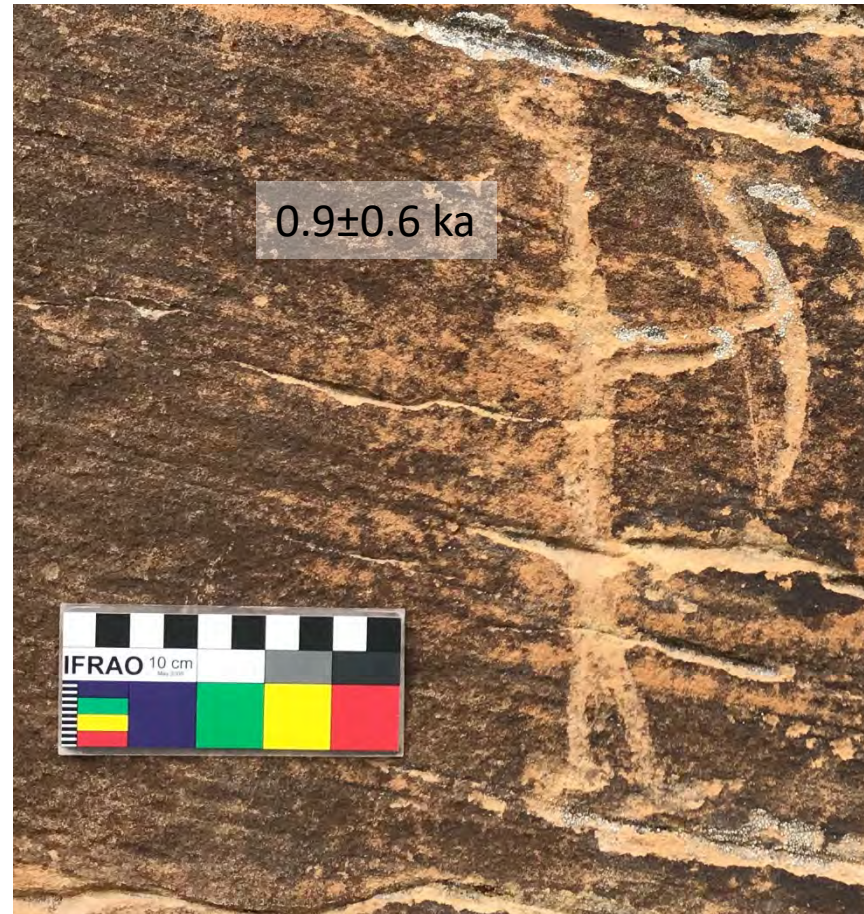

PC2508

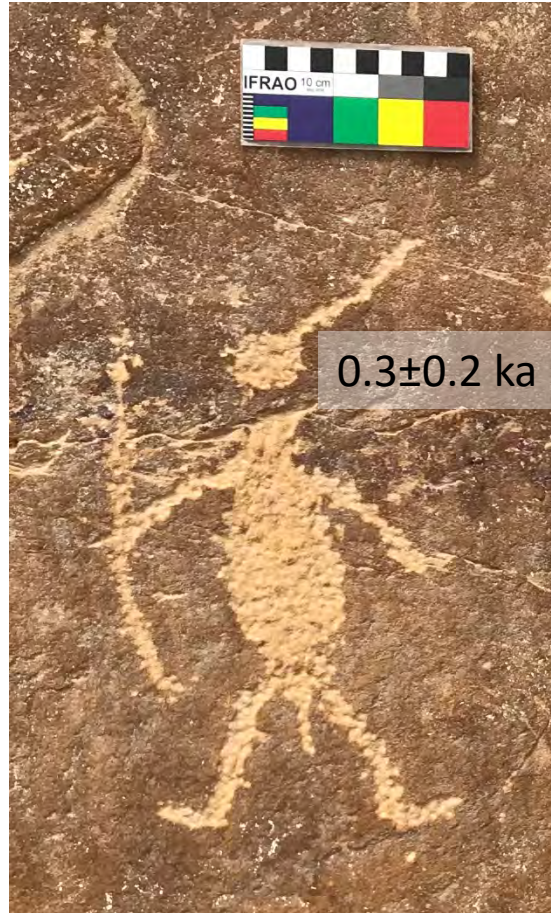

Supplement: S4 File — (PDF) [file pone.0263189.s005.pdf]
