# Supplementary material for: Archaeometric studies on rock art at four sites in the northeastern Great Basin of North America
Source: PLoS One. 2022 Jan 26;17(1):e0263189. doi: 10.1371/journal.pone.0263189 (PMC8791535; doi:10.1371/journal.pone.0263189)
Supplement: Attachment — Submitted filename: Response_to_Reviewers_PONE-D-21-23506.docx [file pone.0263189.s006.docx]

Our responses to the reviewers’ comments are given below. (The reviewers’ comments are in italics).

**Reviewer #1:**

*Nice work. In particular, the authors fully described the pitfalls of the technique. Might help to give a better idea about "significant uncertainty" in absolute dating. I see two types of error - things gone completely wrong and things working, subject to the many expected small perturbations in conditions.*

We added the following text to the Summary and Conclusions:

“Cases with very large errors might be identified by obvious age disagreements between iconographically similar images, by age estimates outside of the archaeologically possible range, or from strong disagreement between ages based on absolute and normalized Mn densities (e.g., MR2053, Fig 6). The effect of smaller errors, on the other hand, can be mitigated by increasing the number of petroglyphs analyzed and focusing more on the ages obtained for groups of iconographically related elements than for individual images.” *I have a bit of concern about their use of the calibration curve from the cathedral study [83]. Were their varnish thicknesses and Mn content similar to those in that study?*

Actually, the calibration samples in the “cathedral study” (Macholdt et al., 2017a) were not samples of the varnish from the cathedral, but varnish-coated rock samples from the California desert, with a composition typical of the rock varnish found in the western US. The Mn areal densities on these calibration samples (100 – 700 µg cm^-2^) is comparable to the densities of the varnish in our present study (0 – 910 µg cm^-2^). *Also, did the non-linear attenuation for the rock Mn-content come into play with the straight rock background correction?*

The non-linearity due to absorption of the incoming and outgoing radiation by the sample is taken into account by the instrument software. This assumes, however, a homogeneous sample. In the case of a Fe- and Mn-rich varnish on top of the bare rock, attenuation in the varnish would reduce the signal from the underlying rock, leading to a lower contribution of the Mn in the rock to the total signal. This would result in a too large background correction, if the value from the bare rock is used.

We ignored this non-linearity for the following reasons. In the case of the sandstones, the background correction is very small, usually less than 2 µg cm^-2^. The nonlinearity would only become important for very thick varnish layers (hundreds of µg cm^-2^). For example, our measurements on calibration samples did not show evidence for nonlinearity for varnish thicknesses up to 700 µg cm^-2^ (Macholdt et al., 2017a). In this case the small uncertainty due to the nonlinearity of the signal would be irrelevant compared to the other sources of variability and error. Even the case of the basalts, where the background can contribute as much as the equivalent of 30 µg cm^-2^, the potential errors at high varnish thicknesses would be minor compared to the spatial variability of the measurements (ca. 24%) and the uncertainty in the Mn deposition rate.

**Reviewer #2:**

*This paper proposes a method to date ancient petroglyphs based on measurements by p-XRF of the areal densities on manganese and iron. The idea is based on the concept that the amount of varnish that has regrown on a petroglyph since its creation, relative to the surrounding intact varnish, is a measure of its age.

It seems a promising manuscript from the title and abstract, but the content lacks rigour since there are statements that evidence authors’ poor knowledge of XRF analysis. The method has been already published ([46, 47]).*

Exactly! This is not a methodological paper, but one where an established and multiply peer-review technique is applied to new study sites. We do take exception to the insulting statement about the “authors’ poor knowledge”. It is inappropriate for a reviewer to insult the authors.

*Moreover, the experimentation is very poor: using a single spectroscopic technique is insufficient to characterize a material.*

We feel this criticism (“the experimentation is very poor”) is inappropriate. We would never claim that “using a single spectroscopic technique is sufficient to characterize a material”. In fact, we have published numerous papers where we apply multiple and highly sophisticated techniques to characterize rock varnish (Macholdt et al., 2015; Otter et al., 2015 ; Macholdt et al., 2017a; Macholdt et al., 2017b; Lang-Yona et al., 2018; Macholdt et al., 2019; Förster et al., 2021). Here, we focus on a single property, the areal density of Mn in rock varnish, for which our technique is highly appropriate and sufficient. Note also, that all the other techniques, which would not provide additional information relevant to rock art dating, require destructive sampling, which is not desirable for archaeological materials.

*The authors they-self state : “We emphasize that these approaches remain experimental and have to be viewed with great caution, because the growth rate of varnish is highly variable and depends on a large number of parameters other than age, including the exposure of the rock surface to dust, erosion by wind and water, the orientation and slope of the rock surface, the hardness, roughness, and texture of the rock underneath, and its initial iron content, as summarized in Andreae, Al-Amri [7, and references therein]”. Because all of this, I recommend rejecting this paper.*

Note that other reviewers (e.g., R1, but also reviewers of our other papers) have praised our honest discussions of the potential sources of error and pitfalls in this type of work. But rock art dating is an extremely difficult problem because of the absence of reliable radiometric tracers in petroglyph material. If techniques like **^14^C** dating were possible, there would be no reason to use a technique such as ours. But in the absence of such techniques, our method at least provides age estimates albeit with admittedly large error bars. We make this very clear in our paper. Furthermore, we use the normalized Mn density, N_Mn_, which compensates for some of these non-age-related factors. *My specific concerns are the following:

1) Introduction Section: It is hard to find out how this paper is advancing the state-of-the-art since it is not explicitly clarified in the paper. I suggest the authors dedicate a section to explain this.*

We added the following statement at the end of the Introduction:

“Our study extends significantly the very scarce knowledge base on the rate of rock varnish accumulation in semiarid to mesic regions, provides further validation of a non-destructive dating technique for rock art, and elucidates the history of rock art creation at four important archaeological sites representing the Great Basin Macrotradition.”

Note that this is only the second paper on the use of this technique to date rock art in all of North America! *2) Material and Methods Section: why the four archaeological sites have been chosen is not provided?*

This information is provided in the Introduction section:

“This region was chosen because it contains a great variety of rock art ranging in age from Paleoindian to Historic times, and because the availability of previously dated rock art allows a comparison of these dates with our experimental technique (Francis, 2018). In addition, the presence of dated Holocene lava flows (Putirka et al., 2009) and boulders that were abraded during a massive flood event at 14.5 ka BP (Pavesic, 2007) in the Snake River Valley provides an opportunity to assess the rate of varnish formation in this area.”

*The sentences “The pXRF measurements were conducted using a Niton XL3 pXRF (Thermo Fisher Scientific) in the “mining” mode” and “The pXRF measurements, reported as percentage by the instrument” are unacceptable in a scientific paper.*

Note that we kept the Methods section deliberately short, since this method has been used in several previous papers, and has been peer-reviewed by some ten reviewers, including in PLOS-ONE. The “Mining” mode is simply the name used in the instrument software for the analysis of geochemical samples, and the instrument software does report the sample composition as mass percent. In previous work, we have shown that the percentage values reported by the instrument software provides identical results to those obtained by the analysis of the X-ray fluorescence spectra (Macholdt et al., 2017a).

To make this more transparent to readers, we have made some modifications to the text:

“The pXRF measurements were conducted using a Niton XL3 pXRF (Thermo Fisher Scientific) in the “mining” mode, which refers to the internal software setting appropriate for geochemical samples.”

“The pXRF measurements, reported by the instrument as mass percentages of elements in the rock sample, were converted into areal density values …”

*Finally, I have observed that most of the methods used are not developed by the authors but taken from the literature. Specifically, this method is not new (others applied it before [46, 47]). Moreover, the p-XRF method is not improved in this paper but applied to solve their problem. So there is no scientific contribution in this aspect either.*

We make no claim that this paper describes a novel technique or a technical development. Quite the contrary, we are using an established and multiply peer-reviewed technique to obtain novel geochemical (varnish growth rates) and archaeological results.

*As for the archaeological, I’m not able to judge if the results are worth to be published. Surely, whatever the historical and artistic repercussions, the authors must be sure that the experimental data on which their considerations are based are valid. This is not the case.*

Given the extensive prior peer review of the methods, and our substantial experience with its application, I see no justification for questioning the validity of the data. Notably, the reviewer provides no evidence for his claim that the data are not valid.

**Reviewer #3:**

*In this paper, the possibility of deriving age estimates from measurements of the areal density of manganese (DMn) and iron (DFe) in rock paint on petroglyphs was explored. It is based on the fact that the amount of paint that has grown back on a petroglyph since its creation, relative to the surrounding intact paint, is a measure of its age. To determine the areal densities of Mn (DMn) and Fe (DFe) in the rock paint, XRF measurements were conducted with a hand-held spectrometer on petroglyphs located in the Snake River Valley of southern Idaho and the Wind River Basin of western Wyoming and adjacent to southern Montana. In addition, XRF measurements were made on the intact paint surrounding rock art to assess the variability of paint formation between different rock surfaces and on standards (rock surfaces of known geological age) to assess their potential to derive accumulation rates of paint. In conclusion, the age estimates of the petroglyphs were derived and evaluated in their archaeological and cultural context. The results, in most cases, are in good agreement with chronologies based on archaeological and other archaeometric techniques.
The authors are clearly aware of the limitations of the method and the results, but explain the reasons effectively. This article could be a starting point for non-destructively dating important historical artifacts.

The experiments, statistics and other analyzes performed are described in sufficient detail. The sample size (461 measurements) is adequate to produce robust results. While the method is subject to significant uncertainty regarding the absolute age of individual images.
The conclusions are presented appropriately and are supported by the data. Where this was not possible, a reasonable discussion was reported.
The article is presented in an understandable way and is written in standard English.

The authors are not new to this field; in previous work on the paint of rocks in Arabia and North America, they showed that the age estimates obtained (determining the areal density of Mn (DMn) and Fe (DFe)) were consistent with the ages based on the cultural and ecological content of the art rock and allowed a significant ordering of the rock images in a sequence of ages. Therefore, the presented study replicates and is very similar to the previous work, but the authors provided solid scientific rationale for the presented work by clearly referencing and discussing existing literature.

In conclusion, in my opinion the document is suitable for publication and requires only a minor revision.

Specific comments:
The materials part should be reduced.*

The material section is admittedly quite detailed. However, it was written after extensive discussions with our archaeologist colleagues, who felt that in view of the confused and often contradictory classification and nomenclature of Big Basin rock art it was essential to provide a complete and specific discussion of the archaeological context of this work. Therefore, we would prefer not to remove material from this section.

*The pXRF instrumentation part should be better described. For example, what it means "Filter passes and integration periods were:" standard "25 s," low "15 s," high "20 s, and" light "25 s." (lines 300 and 301) What's the difference?*

As mentioned above, we felt that the methods section should be very brief, since the technical parts of this work have been described in detail in several previous papers. The instrument used a sequence of filter foils, which select the appropriate energy ranges of the X-rays for the different elements analyzed. Low, High, and Light refers to the atomic number of the elements for which the filters are optimal, and the time with each filter indicates the length of time that the measurements are performed and integrated with each filter. We have added a little bit of text here, but since this is quite standard for this type of instrument, we did not want to go into lengthy explanations.

“During the measurement cycle, a series of filters are placed in the path of the X-rays and the anode voltage is adjusted to be optimal for elements with different atomic number, Z. The filter steps and integration periods over which measurements were made with each filter setting were: “Standard”: 25 s, “Low-Z”: 15 s, “High-Z”: 20 s, and “Light Elements”: 25 s.” *Line 385 The value on this flow surface is 0.81 ± 32, is the error correct?*

Corrected to 0.32

*Line 809 “rack varnish” correct it into “rock varnish”.*

Done.

References:

Förster, J.-D., Bykova, I., Macholdt, D. S., Jochum, K. P., Kappl, M., Kilcoyne, D. L., Müller, M., Sorowka, A., Weber, B., Weigand, M., Schütz, G., Andreae, M. O., and Pöhlker, C., X-ray microspectroscopy and ptychography on nanoscale structures in rock varnish: ACS Nano, under review, 2021.

Francis, J. E., Age estimates and classification of pecked rock art, in *Dinwoody Dissected: Looking at the Interrelationships Between Central Wyoming Petroglyphs*, edited by D. N. Walker, pp. 159-184, 2018.

Lang-Yona, N., Maier, S., Macholdt, D. S., Mueller-Germann, I., Yordanova, P., Rodriguez-Caballero, E., Jochum, K. P., Al-Amri, A., Andreae, M. O., Froehlich-Nowoisky, J., and Weber, B., Insights into microbial involvement in desert varnish formation retrieved from metagenomic analysis: Environmental Microbiology Reports, 10, 264-271, doi:10.1111/1758-2229.12634, 2018.

Macholdt, D. S., Jochum, K. P., Pöhlker, C., Stoll, B., Weis, U., Weber, B., Müller, M., Kappl, M., Buhre, S., Kilcoyne, A. L. D., Weigand, M., Scholz, D., Al-Amri, A. M., and M. O. Andreae, Microanalytical methods for in-situ high-resolution analysis of rock varnish at the micrometer to nanometer scale: Chemical Geology, 411, 57-68, doi:10.1016/j.chemgeo.2015.06.023, 2015.

Macholdt, D. S., Herrmann, S., Jochum, K. P., Kilcoyne, A. L. D., Laubscher, T., Pfisterer, J. H. K., Pöhlker, C., Schwager, B., Weber, B., Weigand, M., Domke, K. F., and Andreae, M. O., Black manganese-rich crusts on a Gothic cathedral: Atmospheric Environment, 171, 205-220, doi:10.1016/j.atmosenv.2017.10.022, 2017a.

Macholdt, D. S., Jochum, K. P., Pöhlker, C., Arangio, A., Förster, J. D., Stoll, B., Weis, U., Weber, B., Müller, M., Kappl, M., Shiraiwa, M., Kilcoyne, A. L. D., Weigand, M., Scholz, D., Haug, G. H., Al-Amri, A., and Andreae, M. O., Characterization and differentiation of rock varnish types from different environments by microanalytical techniques: Chemical Geology, 459, 91-118, doi:10.1016/j.chemgeo.2017.04.009, 2017b.

Macholdt, D. S., Förster, J. D., Müller, M., Weber, B., Kappl, M., Kilcoyne, A. L. D., Weigand, M., Leitner, J., Jochum, K. P., Pöhlker, C., and Andreae, M. O., Artifacts from manganese reduction in rock samples prepared by focused ion beam (FIB) slicing for X-ray microspectroscopy: Geosci. Instrum. Method. Data Syst., 8, 97-111, doi:10.5194/gi-8-97-2019, 2019.

Otter, L., Macholdt, D., Jochum, K., Stoll, B., Weis, U., Weber, B., Scholz, D., Al-Amri, A., and Andreae, M., Desert varnish and dust: Nano- and femtosecond LA-ICP-MS studies of major and trace elements: Goldschmidt Abstracts, 2367, 2015

Pavesic, M. G., The Bonneville flood debris field as sacred landscape: Journal of California and Great Basin Anthropology, 27, 15-27, 2007.

Putirka, K. D., Kuntz, M. A., Unruh, D. M., and Vaid, N., Magma evolution and ascent at the Craters of the Moon and neighboring volcanic fields, southern Idaho, USA: Implications for the evolution of polygenetic and monogenetic volcanic fields: Journal of Petrology, 50, 1639-1665, doi:10.1093/petrology/egp045, 2009.
